# The Guanacaste Volcanic Arc Sliver of Northwestern Costa Rica

**DOI:** 10.1038/s41598-017-01593-8

**Published:** 2017-05-11

**Authors:** Walter Montero, Jonathan C. Lewis, Maria Cristina Araya

**Affiliations:** 1 Centro de Investigaciones en Ciencias Geológicas, Universidad de Costa Rica, San Pedro, Costa Rica; 2 114 Walsh Hall, Geoscience Department, Indiana University of Pennsylvania, Indiana, PA 15705 USA; 3 Red Sismológica Nacional (RSN: UCR-ICE), Apdo. 214-2060, Escuela Centroamericana de Geología, Universidad de Costa Rica, San Pedro, Costa Rica

## Abstract

Recent studies have shown that the Nicoya Peninsula of northwestern Costa Rica is moving northwestward ~11 mm a^−1^ as part of a tectonic sliver. Toward the northwest in El Salvador the northern sliver boundary is marked by a dextral strike-slip fault system active since Late Pleistocene time. To the southeast there is no consensus on what constitutes the northern boundary of the sliver, although a system of active crustal faults has been described in central Costa Rica. Here we propose that the Haciendas-Chiripa fault system serves as the northeastern boundary for the sliver and that the sliver includes most of the Guanacaste volcanic arc, herein the Guanacaste Volcanic Arc Sliver. In this paper we provide constraints on the geometry and kinematics of the boundary of the Guanacaste Volcanic Arc Sliver that are timely and essential to any models aimed at resolving the driving mechanism for sliver motion. Our results are also critical for assessing geological hazards in northwestern Costa Rica.

## Introduction

Ever since forearc slivers were first identified^1^ considerable attention has been paid to the impact that sliver motion has on plate motion budgets^2^ and forearc deformation^3, 4^, but less attention has focused on the geology of their trailing edges^5–7^. Geodetic work has demonstrated that in northwestern Costa Rica, the Nicoya Peninsula is moving northwestward ~11 mm a^−1^ relative to eastern Costa Rica^8–11^. Forearc motion in El Salvador, to the northwest, is accommodated by a well characterized dextral strike-slip fault system^9, 12, 13^. The configuration of the sliver to the southeast in the more populous parts of Costa Rica has to date remained unresolved. As a consequence, the scale of the tectonic sliver and the potential roles that it might play in the tectonic, seismologic and volcanic history of Costa Rica are poorly understood. Here we document a fault system that we suggest serves as the northeastern margin of the sliver, and that traverses the Guanacaste volcanic arc. The configuration of this tectonic boundary, herein referred to as the Haciendas-Chiripa fault system, requires that what has previously been described as a forearc sliver^10^ includes most of the Guanacaste volcanic arc. By documenting the geometry and kinematics of this fault system on the margin of the Guanacaste Volcanic Arc Sliver (GVAS) we provide constraints that are essential for models of sliver motion that aim to disentangle the relative contributions of ridge collision^5, 10^, oblique subduction^2^ or trailing edge plate forces^14^.

## Tectonic Setting

**Figure 1:**
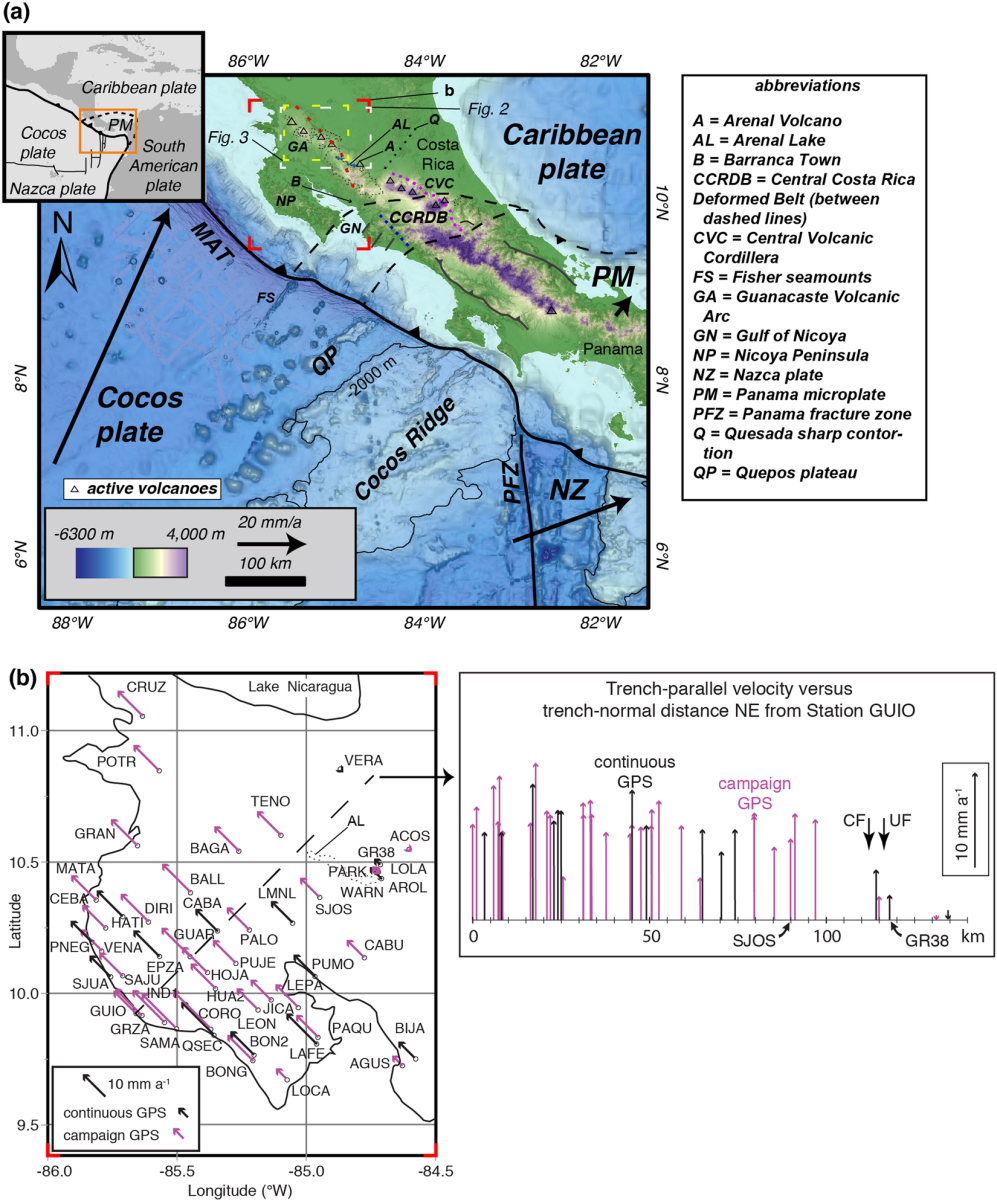
(**a**) Tectonic setting of Costa Rica. Topographic and bathymetric base map simplified from Morell^31^. Dotted outlines are Monteverde arc of Carr *et al*.^18^. Location of the Quesada Sharp Contortion from Protti *et al*.^29^. Red dashed line outlines the Haciendas-Chiripa fault system. The dashed magenta and blue lines show the positions of the San Miguel-Atirro-Rio Sucio dextral fault system^6^ and the Candelaria fault^6^, respectively. (**b**) Trench-parallel component of GPS velocities from Feng *et al*.^8^. Magenta vectors are from campaign GPS and black vectors are for permanent GPS stations. The profile at the upper right of (**b**) shows the same velocity plotted versus distance perpendicular to the trench. UF = Upala Fault and CF = Chiquero Fault. The location of Fig. 1b is indicated by red corners. The location of Figs 2 and 3 are shown by the dashed white and yellow boxes, respectively. Map at (**a**) and inset were provided with permission to modify by K. Morell and modified using Adobe Illustrator CC 2015 release. Map and plot at (**b**) were generated using Adobe Illustrator CC 2015 release.

Central Costa Rica occupies the southern boundary of the Caribbean plate (CA) where it is in contact with the Panama microplate, and is bounded to the southwest by the Middle America Trench (MAT, Fig. 1). The broad, concave-to-the-SE zone of diffuse deformation that separates CA and Panama microplate is the Central Costa Rica Deformed Belt (CCRDB)^15, 16^. Widespread mesoscale faulting in the CCRDB has been attributed to the subduction of rough seafloor at the MAT^16^. This interpretation is in agreement with the observation that the western edge of the CCRDB meets the Pacific coast at the northeast-trending boundary on the downgoing Cocos plate that separates smooth seafloor to the northwest from rough seafloor to the southeast^17, 18^. In western Costa Rica the Cocos plate is subducting toward the northeast at ~81 mm a^−1^ relative to CA, and at a high angle to the MAT^2^. Recent mapping suggests that locally the CCRDB is being crosscut by the dextral Atirro-Río Sucio fault system that accommodates lateral extrusion of the upper plate in response to Cocos Ridge collision^6^.

Northwestern Costa Rica is dominated by the northwest-trending, extinct (~1.0–2.2 Ma) Monteverde volcanic arc, and the active Guanacaste volcanic arc (Fig. 1a) reflecting subduction of the Cocos plate^19^. The volcanic-arc rocks are locally covered by epiclastic and alluvial deposits on both the southern and northern flanks of the arc^19^. At the Pacific coast, the Nicoya Peninsula and coastal areas to the east (across the Gulf of Nicoya) expose oceanic basement and cover sediments of fluvial and marine origin^15, 20^. The Costa Rican forearc appears to reflect the influence of lower plate architecture. For example, immediately inboard of the Quepos Plateau (Fig. 1) the rocks record differential tilting, associated mostly with NE-striking faults, and this has produced asymmetric watersheds^21^. This influence ceases toward the northwest at approximately the Gulf of Nicoya^21^, with the exception of the southernmost tip of the Nicoya Peninsula where the incoming Fisher Seamount Chain drives high Holocene uplift rates (~6 m ka^−1^) and northward tilting^22^.

## Results

On the basis of existing GPS velocities, well-located earthquake hypocenters, new focal mechanism solutions, and field observations we document the northeastern boundary of the GVAS from the area south of Lake Arenal northwestward ~100 km. We infer based on GPS velocities (Fig. 1b) that the fault system can be traced from the Lake Arenal area southeastward to the Gulf of Nicoya coastal area near Barranca town (see B in Fig. 1a). We refer to this boundary as the Haciendas-Chiripa fault system. GPS geodesy data provide critical, but yet incomplete, constraints on the boundary between the GVAS and the rest of Central America. Recent GPS work^8^ showed that the Nicoya Peninsula is moving essentially as a rigid block, and that the trench-parallel component of motion diminishes significantly eastward across the Gulf of Nicoya and the area of Lake Arenal (Fig. 1b). Northeastward across the active volcanic arc the number of GPS stations is limited, however station VERA located south of Lake Nicaragua records no discernable trench-parallel motion, suggesting that the GVAS boundary occurs toward the southwest between this station and station TENO. Similar changes in velocity are noted in the area of Lake Arenal, for example a substantial reduction in velocity is observed northeastward from station SJOS toward station GR38 (Fig. 1b upper right). In the following paragraphs we present evidence for active deformation that is most easily understood by invoking a through-going fault system that functions as the northeastern boundary of the GVAS.

**Figure 2:**
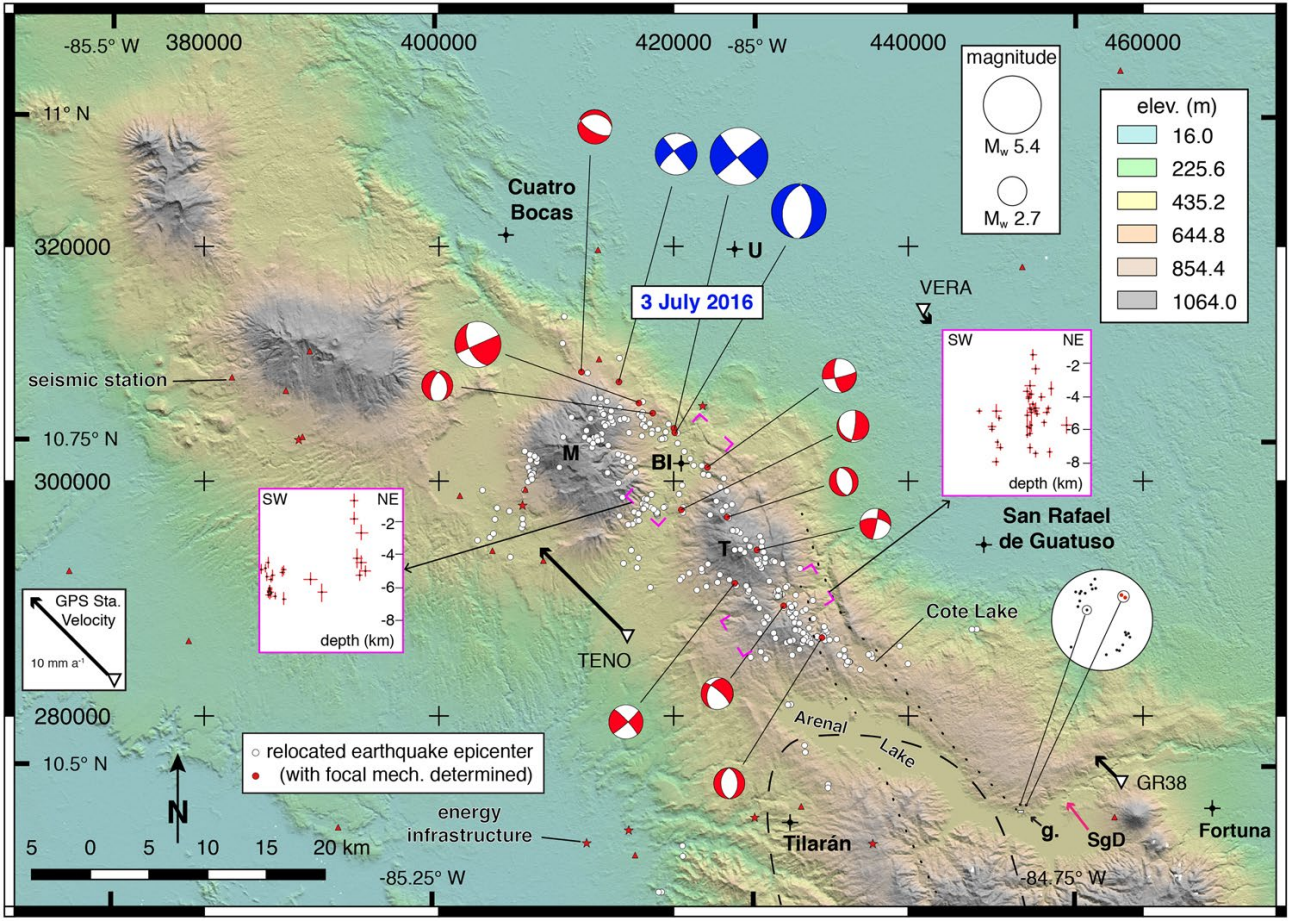
Seismotectonic setting. Geographic reference system here and in Fig. 3 is that found on most topographic maps of Costa Rica, the Ocotepeque 1935/Costa Rica Sur. Latitude and longitude graticules using the WGS 84 reference frame also shown here and in Fig. 3. Digital elevation model and shaded relief map derived from enhanced Shuttle Radar Topography Mission (SRTM) data with an effective resolution of 30 m. Lighting from 020°. Lower-case bold letter g. with view direction for photograph at Fig. 4g. The stereonet shows poles to faults at the outcrop at location g. The red poles show the geometry of the bounding strike-slip fault, the principal displacement zone^27^ at the eastern end of the exposure (right end of white symbol), whereas the black poles show normal faults that record dip separations that average ~0.6 m. The circled black pole is the westernmost fault. Portions of the Cote-Arenal fault and Chiripa faults from Fig. 3 are included as a dotted line. The dashed line outlines the aftershock area for the 13 April 1973 M_s_6.5 Tilarán earthquake^29^. The dashed magenta boxes show the locations of the two inset seismicity profiles in which the uncertainty in the earthquake locations is shown by vertical and horizontal red lines. Towns are shown with a dot-plus symbol. Town abbreviations: BI = Bijagua and U = Upala. Volcanoes: M = Miravalles and T = Tenorio. SgD with red arrow = Sangregado Dam. Map generated using QGIS version 2.14 with SRTM 1 Arc-Second Void Filled digital elevation data courtesy of the U.S. Geological Survey from https://earthexplorer.usgs.gov/. Keys, labels and symbols were added using Adobe Illustrator CC 2015 release.

Well-located crustal earthquakes and focal mechanism solutions provide robust constraints on the northeastern GVAS boundary (Figs 1a and 2). We have taken advantage of an improved crustal 1D velocity model for western Costa Rica^23^ (±0.147 km in latitude, ±0.072 km in longitude and ±0.074 km in depth) and used HypoDD^24^ to locate earthquakes precisely and these define a seismic source zone where field observations confirm evidence of active or recent faulting. In Fig. 2 we show locations for earthquakes recorded by a local network from December 14, 2006 until July 29, 2016. These include three recent moderate magnitude events on July 3, 2016 that reveal strike slip motion and normal motion (Fig. 2). Our new focal mechanisms are consistent with historical events included in the Centroid Moment Tensor (CMT) catalog.

**Figure 3:**
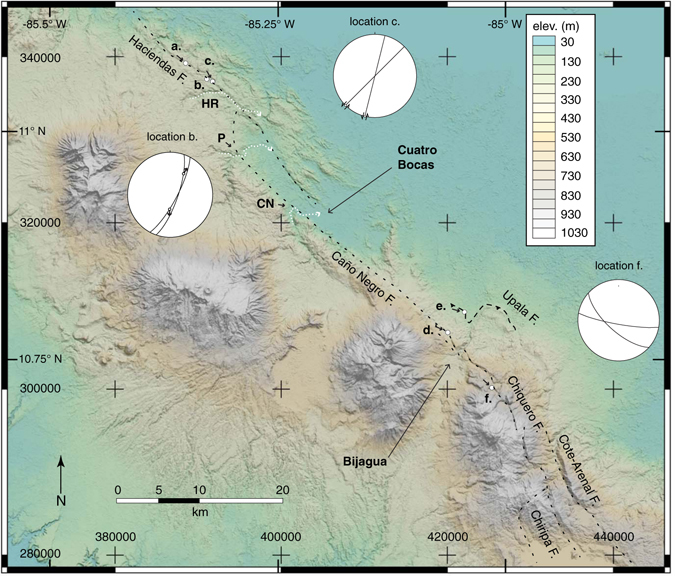
Haciendas-Chiripa fault system. Digital elevation model and shaded relief map derived from enhanced SRTM data with an effective resolution of 30 m. Lighting is directed from an azimuth of 020°. Mapped fault segments are named and the locations of the major fault traces are shown with dashed lines. Lower-case bold letters a.–f. with short arrows showing view direction of lettered photos at Fig. 4. Lower-hemisphere equal-area projections show structural data described in text. CN = Caño Negro River, HR = Haciendas River and P = Pizote River. Map generated using QGIS version 2.14 with SRTM 1 Arc-Second Void Filled digital elevation data courtesy of the U.S. Geological Survey from https://earthexplorer.usgs.gov/. Keys, labels and symbols were added using Adobe Illustrator CC 2015 release.

**Figure 4:**
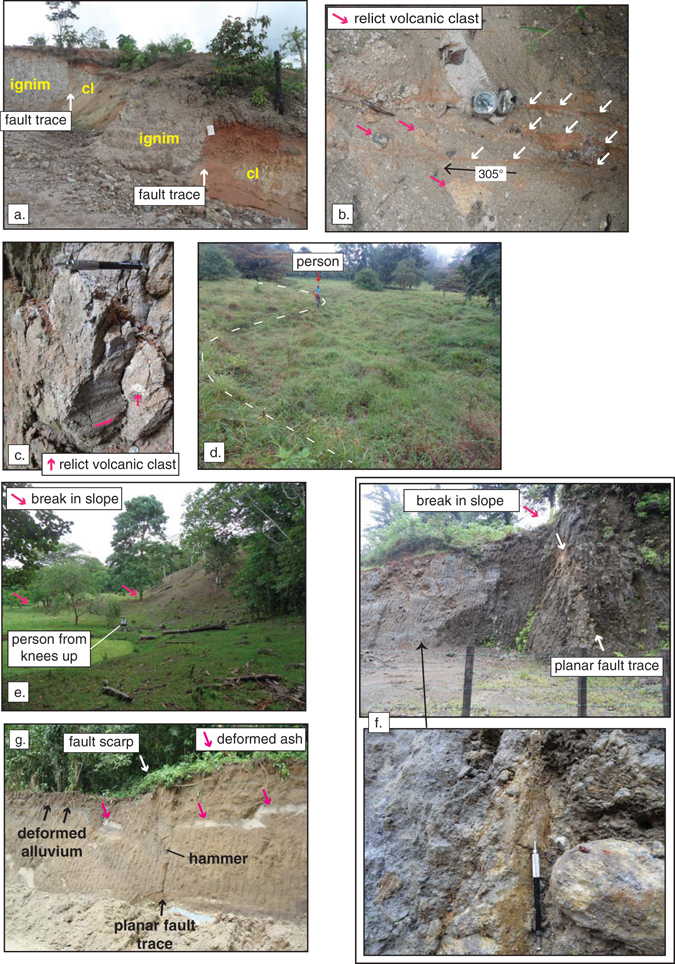
Field photographs. Locations as lower-case bold letters in Figs 2 and 3. (**a**) Fault trace where it juxtaposes fresh ignimbrite (ignim) against clay gouge (cl). White fieldbook is 14 cm in width. (**b**) Looking down on ignimbrite with thin, ~vertical clay seams that strike ~305°. White arrows show three seams. Pocket transit 7 cm wide. (**c**) Cutbank exposure of fault gouge containing anastomosing vertical subsidiary shear planes, slip shown by magenta half arrow. (**d**) Drainage deflected ~5.5 m dextrally within a small pull-apart basin. Dashed white lines highlight the curvature of the margins of the stream. (**e**) Thrust scarp in the Upala fault segment. (**f**) Planar, steeply dipping fault trace in deeply weathered exposure on the road to Tenorio Volcano. Detail at bottom shows steeply raking, subtle lineations (~parallel to pencil) on a polished fault plane preserved in deeply weathered, clay rich fault rock. (**g**) Fault exposed in an ephemeral outcrop that extended >120 m west from this location along the shores of Lake Arenal in 2008. The poles to fault planes observed in this outcrop are shown in the stereonet in Fig. 2. Photographs annotated using Adobe Illustrator CC 2015 release.

Field observations coupled with analyses of aerial photos and Digital Elevation Models (DEMs) reveal evidence of recent or active faulting along the Haciendas-Chiripa fault system. The northern flank of the Guanacaste volcanic arc in northwestern Costa Rica is marked by a clear topographic lineament (Fig. 3) that includes fault exposures and well-preserved fault scarps (Fig. 4). Fresh outcrops can be found at quarries and roadcuts, but owing to the humid tropical climate they tend to be ephemeral. This is in fact one of the major challenges to mapping active or recently active faults in Central America. Along streams most bedrock exposures are deeply weathered, however, lithologies and primary structures are sometimes preserved. At location **a** (Fig. 3) there is a recent, irregular roadcut that exposes the fault trace at two locations separated by several meters (Fig. 4a). The steeply dipping fault juxtaposes relatively fresh ignimbrite to the northeast with a clayey unit to the southwest that we interpret as fault gouge. The trend of the fault trace where it is cut by the excavation is ~295° and based on the topographic lineament we infer a fault strike of ~300° in this area. We refer to this as the Haciendas fault segment. The presence of the fault is highlighted here by the lateral change in soil character. Above the ignimbrite is a dm-scale reddish brown soil horizon whereas above the gouge is a 0.5 m thick, bright red lateritic soil. The ignimbrite includes recognizable, fresh volcanic lithic fragments of varying size whereas the clay gouge unit consists of a deeply weathered matrix of highly plastic clay containing small relict volcanic clasts.

Along strike to the southeast at location **b** (Fig. 3) there is a very similar roadcut through an elongate E-W ignimbrite unit that is truncated on its eastern end by the topographic lineament that we map as the Haciendas fault. The main body of the ignimbrite is cut by numerous conjugate, m-scale normal faults that record minor offsets (dm-scale). Many of these faults cut the entire thickness of the unit, and although kinematic indicators are rarely preserved, overall they suggest ~E-W stretching (see station b stereonet at Fig. 3). The eastern end of the ignimbrite, within ~2 meters of where it is truncated, is cut by numerous, cm-scale, sub-vertical anastomosing clay seams that trend west northwest (Fig. 4b). We interpret these to be minor shear zones that reflect damage-zone processes adjacent to the mature Haciendas fault. Downslope and to the east at location **c** is a stream cutbank exposure of deeply weathered clayey ignimbrite that is cut by anastomosing sub-vertical, lineated antithetic subsidiary (Riedel) shear planes (Fig. 4c). This exposure is along strike from location **a** described above and we interpret it to lie within the fault zone. Immediately to south is the Haciendas River which shows a strong right deflection (see HR in Fig. 3).

Southeast from the Haciendas fault we trace additional fault segments based on topographic expression, offset drainages, exposures of highly sheared rock, and seismicity. These fault segments are from west to east the Caño Negro, Upala, Chiquero, Cote-Arenal and Chiripa. In the Caño Negro segment the topographic lineament bifurcates where the fault displays a right (releasing) step (Fig. 3). Near the town of Cuatro Bocas, there are additional abundant deflected streams and local exposures of highly sheared Quaternary volcanic strata that mark the fault trace. Terrace risers where the Pizote and Caño Negro Rivers cross the fault trace display right-deflections on the scale of hundreds of meters (see P and N in Fig. 3).

In the eastern sector, the Caño Negro fault is marked by geomorphic expressions of active or recent slip. North of the town of Bijagua (location **d** in Fig. 3) two fault strands define a narrow, km-scale pull-apart basin that hosts parallel, right-deflected headwater drainages (Fig. 4d). The deflections are sharp and aligned, forming a linear array on the north margin of the pull-apart basin that can be traced for more than 440 meters. We measured a dextral offset of ~5.5 m at location **d** (Fig. 4d). To the east southeast the fault is apparent as a strong topographic lineament on the north flank of Tenorio Volcano (T in Fig. 2). We have generated new focal mechanisms for three moderate magnitude earthquakes that occurred near Bijagua on 3 July 2016 (Fig. 2), two revealing strike-slip faulting and one showing normal faulting. All three events indicate approximately E-W extension. Importantly, <10 km to the northeast a M_w_ 5.4 thrust earthquake occurred on 27 January 2002^25^ and we have identified the probable scarp at location **e** (Fig. 4e). We map this structure as the Upala fault. The scarp occurs on the northern edge of a landscape that: (1) includes surface slopes that are tilted southward, toward the edifices of Tenorio and Miravalles volcanoes; and (2) hosts alluvial sediments and small meandering streams in an area otherwise dominated by higher stream gradients. Exposures of bedrock in a stream that has incised the hangingwall of the Upala fault includes an ignimbrite unit with a dip angle >70°, attesting to significant tilting (for example near location **e**). A local resident reported to us in June of 2016 that he noted changes to the landscape in the immediate aftermath of the 2002 earthquake that are consistent with overall northward motion of the hangingwall – specifically open cracks parallel to the fault trace. The occurrence of thrusting north of the main trace of the Caño Negro segment suggests local partitioning of slip within the fault system.

Southeast from Bijagua town the fault system curves slightly taking on a more south southeast trend in the subparallel Chiquero, Cote-Arenal and Chiripa segments. Clear fault scarps mark the fault traces and a recent roadcut on the road from Bijagua to the Tenorio Volcano National Park (location **f** in Fig. 3) exposes a zone of localized shearing (Fig. 4f). This outcrop also showcases the deeply weathered nature of the rocks that we suggest mark the locus of deformation in the fault system. The rock here is highly altered to clay minerals and the preservation of fault-kinematic indicators is poor (Fig. 4f bottom image).

Well-located earthquakes (Fig. 2) also provide constraints on the fault system in this area, in particular illuminating the eastern end of the Caño Negro fault from northwest of Bijagua town to the east side of Tenorio Volcano (Figs 2 and 3). Our earthquake catalog covers a relatively short period of time so it is not surprising that some fault scarps do not have co-located epicenters. Northeast-trending seismicity profiles (Fig. 2) show that the earthquakes define steep planes, consistent with our new focal mechanisms, the tectonic setting and the linear surface expression of the fault traces. Most of the hypocenters are deeper than ~2 km, and in spite of small errors in locations, it is not possible to discern details of the fault system architecture at this point. Locally we note offsets between epicenters and fault scarps that highlight the need for more work. Such offsets might reflect the weak mechanical link between fault ruptures at depth and the deeply weathered cover strata, thick soils and relatively steep slopes at the surface. In such a case the overburden may serve to mask or mute the relation between faults and their surface expression. Alternatively, the mismatch is a reflection of an evolving zone of deformation in which slip is not strongly localized and for which the surface traces of additional faults have yet to be identified.

To the southeast, ephemeral outcrops of high strain zones attest to the trace of the Chiquero, Chiripa, and Cote-Arenal segments as they cross Lake Arenal near the Sangregado dam site (location **g** in Fig. 2). In this region the earthquake epicenters tend to be distributed along the mapped fault traces, including a small cluster of events at Cote Lake along strike from where the Cote-Arenal fault traverses the north shore of Lake Arenal. A now-covered exposure west of the Sangregado dam was documented previously^26^, and in 2008 fault data were collected along this >120 m long outcrop. The eastern end of the outcrop is interpreted to be the principal displacement zone (PDZ)^27^ and is marked by the west-facing scarp seen at Fig. 4g and a similarly oriented fault just to the west. These two faults are shown as red poles in the stereonet at Fig. 2. The easternmost of these two faults displaces a paleosol dated at 1370 ± 215 B.C. but with a minimum age estimated at 900 BC^26^. To the west, the outcrop is cut mostly by normal faults oriented at high angles to the PDZ and that record an average of ~0.6 m of dip separation (black poles in stereonet at Fig. 2). The westernmost of these shows 6.35 m of vertical separation and is marked by an east-facing scarp (circled black pole in stereonet in Fig. 2). The geometry and location of the faults are consistent with a local releasing step in the fault system. Importantly, however, ~30 m west of the eastern PDZ a broad antiform occurs in the lower part of the stratigraphic section that is overlain by tephra that is not folded. These observations suggest transient horizontal shortening akin to that observed in other transtensional systems^28^.

South of Lake Arenal the Chiripa segment is marked by linear ridges we interpret to reflect the fault trace (see dotted line south of Lake Arenal in Fig. 2). This region hosted the 13 April 1973 M_s_ 6.5 Tilarán strike slip earthquake^29^ and the aftershocks clustered near the Chiripa fault (Figs 2 and 3). This area also marks a substantial break in GPS velocities (Fig. 1b, see also GPS station trench-parallel velocities for GR38, TENO, and VERA in Fig. 2). The nature of the linkage between these faults and other active structures in eastern Costa Rica remains an important open question. If the faults connect to the San Miguel-Rio Sucio-Atirro fault system north of the Central Volcanic Cordillera (magenta dashed line in Fig. 1a), the geometry implies a regional-scale transpressional (restraining) step. Alternatively, if the faults continue toward the southeast they may link to the Candelaria fault (dark blue dashed line in Fig. 1a) along the Pacific coast.

## Discussion

Our results have immediate implications for hazards mitigation, specifically in the wake of three damaging crustal earthquakes on 3 July 2016 that occurred near Bijagua (Fig. 2). The area that is transected by the Haciendas-Chiripa fault system hosts several important population centers, including Fortuna, Bijagua, Tilarán, San Rafael de Guatuso and Upala (see locations in Figs 2 and 3), as well as numerous hydroelectric projects (see red stars in Fig. 2). Moreover, the volcanic rock that dominates the steep slopes is subject to deep tropical weathering that promotes formation of clay minerals^30^. Many of the ephemeral outcrops in the region expose dm-scale clay horizons that reflect the alteration of volcanic ash to highly plastic bentonite. Mixed with the ignimbrites, tuffs and lava flows that are abundant here are occasional chaotic, block-in-matrix lahar units. All of these lithologies are susceptible to clay alteration rendering them inherently unstable.

Our findings also highlight important targets for future geodetic and geologic work essential to solving fundamental geodynamics questions. Existing GPS data^8^ are inadequate for resolving whether sliver motion is accommodated entirely on the Haciendas-Chiripa fault system. Answering this will require dense GPS transects across the fault, and these would provide constraints on fault locking depths that are essential for evaluating seismic hazards (e.g., estimating maximum moment release). The geodetic results would also highlight targets for complementary geomorphology and paleoseismology investigations. More fundamentally, expanded geodetic work spanning northwestern Costa Rica is essential for addressing the mechanisms that drive sliver motion. Outstanding questions include: (1) When did the sliver form? (2) What is the distribution of slip partitioning on the margins of the sliver? and (3) What is the relationship between the sliver and the segmentation of the volcanic arc? If the sliver reflects only Cocos-ridge collision then it should be no older than the first arrival of the ridge at the MAT ~2–3 Ma^31^. Moreover, the faults accommodating lateral escape might be expected to show an age-progression that reflects northward and radial expansion of the collision zone. This might be reflected by older slip on the Candelaria fault and more recent slip on the San Miguel-Rio Sucio-Atirro fault system (Fig. 1). In contrast, if the sliver is driven by oblique subduction and/or trailing edge plate forces, then the sliver may pre-date Cocos ridge collision. It has been shown through numerical modeling that MAT megathrust coupling is weak^32^, and forearc sliver motion is likely driven by the eastward motion of the Caribbean plate relative to the North American Plate^14^. Such an interpretation is in accord with geodetic modeling results that suggest that oblique convergence is inadequate to explain trench-parallel motion of the Nicaraguan forearc^33^. Understanding the relative contributions of strike slip motion and interplate thrusting will be critical to unraveling the slip budget, and thus important regardless of what drives sliver motion.

With regard to question 3 above, establishing the history of slip on the sliver boundary will also help resolve questions regarding the evolution of volcanism in Central America, including the contributions of upper-plate and lower-plate architecture. Geochronology data (Carr *et al*.^19^) suggest that the current volcanic front of Costa Rica initiated at ~600 Ka, a time of near complete cessation of volcanism in the gap between the Guanacaste Volcanic arc and the Central Volcanic Cordillera (Fig. 1). This gap also marks a poorly understood change from a 100% Ocean Island Basalt (OIB) derived mantle source in the Central Volcanic Cordillera to 20% OIB derived mantle source in Guanacaste arc volcanoes other than Arenal^19^. It is also an area known for the northeast striking Quesada Sharp Contortion (Fig. 1a), which marks a steepening of the Wadati-Benioff zone to the northwest^34^. Recognition that the through-going Haciendas-Chiripa fault system serves as the GVAS boundary in this region provides new constraints with which to explore arc evolution mechanistically.

Lastly, our findings suggest a mechanism for the genesis of allochthonous volcanic terranes. The western cordillera of North America hosts many such terranes that have been attributed to both collisional^35, 36^ and strike-slip tectonics^36–39^. The GVAS therefore provides opportunity to document the lithofacies and structure of a nascent strike-slip allochthonous volcanic arc.

## Methods

All the crustal earthquakes were registered by the Observatorio Sismológico y Vulcanológico Arenal-Miravalles (OSIVAM) from March 2006 through August 2016 (Supplementary Table S1). The earthquakes occurred near or at the volcanic arc and were selected for manual relocation from the seismic network database depending of the following characteristics: a minimum of 8 P wave arrivals at different stations, and with all the evident S wave arrivals at the closest stations from the epicenter, a maximum azimuthal gap of 180° and an initial rms of 1.0 second. The final rms values were consistently much lower, typically ~0.2 or less for the hand-picked events (Supplementary Table S1) and <0.1 for the relocated events (Supplementary Table S2). All the resulting earthquakes are between 1 and 30 km of depth below sea level. The OSIVAM network is an arrangement of stations with Lenartz 3D Lite sensors and Reftek’s 130 s digitizers. The digitizers use a unity gain, and a sample rate of 100 samples per second.

The velocity model used for the manual locations is one-dimensional and consists of 6 layers made for the area and with the station corrections obtained with Velest^23^. After finding the manual locations the events were relocated with a double difference algorithm using HypoDD^24^ (Supplementary Table S2).

The configuration for the double difference relocation was for a medium size dataset, with high certainty of the manual picks. The data used consisted of a catalog of P and S arrival manual picks with a maximum distance of 200 km, with a maximum hypocentral separation of 10 km between events, 10 as maximum number of neighbors per event and a minimum of 5 links to define a neighbor. The initial location is from the catalog and the relocation was resolved in two sets of 5 iterations. The weight for P arrivals was set at one for both sets and a weight of −9 and 0.75 for the S arrivals. The residual threshold in seconds for the first set of iterations was not taken into account and a 5 second threshold for the second set of iterations, and a maximum distance between linked pairs was not restricted for the first iteration and a maximum of 3 km for the second set of iterations. The damping factor used for the first and second iterations was set at 50 and 60, respectively.

Lastly, the new earthquake locations were used to generate focal mechanism solutions. For this we used events with magnitudes greater than M_w_ 3.5 using the program Focmec^40^ and searching every 5 degrees for best solutions (Supplementary Table S2).

## Electronic supplementary material

Supplementary Tables S1 and S2
