# Supplementary material for: The Guanacaste Volcanic Arc Sliver of Northwestern Costa Rica
Source: Sci Rep. 2017 May 11;7:1797. doi: 10.1038/s41598-017-01593-8 (PMC5431924; doi:10.1038/s41598-017-01593-8)
Supplement: Supplementary file 1 — Supplementary Tables S1 and S2 [file 41598_2017_1593_MOESM1_ESM.doc]

Supplementary Tables for:

**The Guanacaste Volcanic Arc Sliver of Northwestern Costa Rica**

Walter Montero^1^, Jonathan C. Lewis^2^ & Maria Cristina Araya^3^

^1^ Centro de Investigaciones en Ciencias Geológicas, Universidad de Costa Rica, San Pedro, Costa Rica. ^2^ 114 Walsh Hall, Geoscience Department, Indiana University of Pennsylvania, Indiana, PA 15705 USA. ^3^ Red Sismológica Nacional (RSN: UCR-ICE), Apdo. 214-2060, Escuela Centroamericana de Geología, Universidad de Costa Rica, San Pedro, Costa Rica. Correspondence and requests for materials should be addressed to J.C.L. (email: jclewis@iup.edu)

Supplementary Table S1. Starting earthquake catalog with manually picked events. The column headings are as follows:

Y = year, M = month, D = day, H = hours, m = minutes and s = seconds, in GMT. LAT = latitude and LON = longitude, in decimal degrees both referenced to the WGS84 reference frame. DEP = depth in km relative to mean sea level. STA = number of seismic stations. RMS = root mean square amplitude. Mag = Moment magnitude (M_w_).

| **Supplementary Table S1** | | | | | | | | | | | |
| --- | --- | --- | --- | --- | --- | --- | --- | --- | --- | --- | --- |
| Y | M | D | H | m | s | LAT | LON | DEP | STA | RMS | Mag |
| 2006 | 12 | 14 | 15 | 4 | 0.2 | 10.624 | -84.996 | 12.5 | 16 | 0.3 | 3.0 |
| 2006 | 12 | 15 | 12 | 51 | 39.4 | 10.624 | -84.993 | 13.9 | 17 | 0.4 | 3.3 |
| 2007 | 12 | 5 | 3 | 27 | 39.6 | 10.650 | -85.057 | 4.2 | 18 | 0.2 | 3.3 |
| 2008 | 4 | 30 | 6 | 20 | 34.3 | 10.614 | -85.003 | 5.6 | 14 | 0.2 | 4.2 |
| 2008 | 5 | 2 | 6 | 31 | 23.2 | 10.622 | -84.974 | 4.7 | 12 | 0.2 | 2.3 |
| 2008 | 12 | 9 | 6 | 36 | 38.3 | 10.603 | -84.941 | 13.1 | 9 | 0.2 | 2.8 |
| 2009 | 5 | 10 | 3 | 19 | 12.7 | 10.709 | -85.102 | 3.5 | 10 | 0.1 | 2.6 |
| 2009 | 6 | 28 | 12 | 13 | 16.0 | 10.671 | -85.089 | 9.1 | 19 | 0.1 | 3.2 |
| 2009 | 8 | 11 | 5 | 9 | 50.8 | 10.570 | -84.960 | 7.6 | 17 | 0.2 | 3.5 |
| 2009 | 8 | 11 | 7 | 43 | 22.3 | 10.567 | -84.964 | 4.8 | 12 | 0.3 | 3.0 |
| 2009 | 8 | 16 | 17 | 51 | 28.6 | 10.641 | -85.003 | 4.7 | 12 | 0.2 | 3.1 |
| 2009 | 10 | 7 | 15 | 29 | 21.7 | 10.562 | -84.906 | 7.5 | 13 | 0.1 | 2.9 |
| 2009 | 10 | 21 | 11 | 4 | 24.3 | 10.592 | -85.012 | 2.2 | 10 | 0.2 | 3.3 |
| 2009 | 10 | 27 | 15 | 59 | 54.4 | 10.615 | -84.977 | 4.6 | 10 | 0.2 | 2.7 |
| 2009 | 11 | 10 | 8 | 30 | 2.4 | 10.683 | -85.029 | 8.3 | 9 | 0.2 | 2.9 |
| 2009 | 12 | 28 | 3 | 44 | 39.8 | 10.621 | -84.980 | 4.7 | 15 | 0.2 | 2.4 |
| 2010 | 3 | 28 | 14 | 23 | 43.5 | 10.680 | -85.017 | 3.1 | 11 | 0.2 | 3.0 |
| 2010 | 7 | 16 | 8 | 12 | 6.9 | 10.650 | -84.970 | 4.5 | 12 | 0.2 | 2.9 |
| 2010 | 7 | 25 | 1 | 50 | 17.2 | 10.602 | -84.987 | 7.1 | 12 | 0.2 | 2.2 |
| 2010 | 10 | 27 | 5 | 15 | 54.0 | 10.662 | -84.994 | 4.3 | 14 | 0.1 | 3.1 |
| 2010 | 11 | 11 | 5 | 2 | 36.2 | 10.703 | -85.023 | 6.0 | 11 | 0.1 | 2.7 |
| 2010 | 12 | 17 | 4 | 56 | 9.9 | 10.709 | -85.080 | 5.7 | 22 | 0.1 | 3.0 |
| 2010 | 12 | 17 | 9 | 5 | 16.8 | 10.704 | -85.076 | 6.2 | 15 | 0.1 | 2.7 |
| 2010 | 12 | 17 | 9 | 8 | 35.6 | 10.699 | -85.075 | 7.3 | 8 | 0.2 | 2.2 |
| 2010 | 12 | 17 | 9 | 9 | 2.3 | 10.679 | -85.086 | 7.9 | 8 | 0.3 | 2.2 |
| 2010 | 12 | 18 | 17 | 53 | 0.8 | 10.680 | -85.082 | 8.8 | 9 | 0.2 | 2.7 |
| 2010 | 12 | 25 | 22 | 59 | 9.9 | 10.688 | -85.075 | 8.3 | 12 | 0.1 | 2.7 |
| 2011 | 1 | 16 | 5 | 8 | 10.1 | 10.712 | -85.085 | 6.4 | 10 | 0.2 | 2.3 |
| 2011 | 5 | 7 | 23 | 22 | 30.7 | 10.642 | -85.012 | 5.4 | 9 | 0.1 | 2.2 |
| 2011 | 5 | 7 | 23 | 25 | 5.3 | 10.645 | -85.012 | 5.0 | 11 | 0.2 | 2.2 |
| 2011 | 5 | 7 | 23 | 40 | 32.1 | 10.636 | -85.010 | 7.0 | 11 | 0.5 | 2.2 |
| 2011 | 5 | 8 | 2 | 7 | 3.1 | 10.642 | -85.013 | 5.7 | 12 | 0.1 | 2.2 |
| 2011 | 5 | 10 | 12 | 13 | 14.2 | 10.599 | -84.966 | 6.9 | 14 | 0.2 | 2.0 |
| 2011 | 5 | 10 | 12 | 27 | 55.4 | 10.594 | -84.972 | 6.7 | 18 | 0.2 | 2.8 |
| 2011 | 5 | 10 | 12 | 47 | 43.1 | 10.604 | -84.964 | 7.1 | 8 | 0.2 | 2.4 |
| 2011 | 5 | 10 | 13 | 3 | 26.4 | 10.592 | -84.969 | 7.3 | 9 | 0.2 | 2.2 |
| 2011 | 5 | 10 | 13 | 29 | 21.6 | 10.600 | -84.965 | 5.9 | 14 | 0.2 | 2.5 |
| 2011 | 5 | 11 | 2 | 29 | 21.1 | 10.598 | -84.967 | 7.4 | 14 | 0.2 | 2.3 |
| 2011 | 5 | 11 | 3 | 21 | 34.3 | 10.598 | -84.961 | 6.6 | 12 | 0.1 | 2.1 |
| 2011 | 5 | 11 | 10 | 44 | 26.9 | 10.573 | -84.915 | 4.0 | 12 | 0.2 | 2.4 |
| 2011 | 5 | 13 | 0 | 11 | 43.6 | 10.621 | -84.962 | 6.8 | 18 | 0.2 | 3.0 |
| 2011 | 5 | 13 | 6 | 12 | 1.1 | 10.597 | -84.972 | 7.2 | 18 | 0.2 | 3.1 |
| 2011 | 5 | 13 | 7 | 59 | 32.6 | 10.602 | -84.970 | 7.5 | 16 | 0.2 | 2.7 |
| 2011 | 5 | 22 | 17 | 16 | 42.1 | 10.602 | -84.968 | 6.5 | 9 | 0.2 | 2.4 |
| 2011 | 5 | 29 | 23 | 1 | 51.6 | 10.657 | -85.090 | 6.8 | 13 | 0.1 | 3.1 |
| 2011 | 6 | 11 | 22 | 55 | 7.2 | 10.688 | -85.083 | 8.1 | 16 | 0.2 | 2.7 |
| 2011 | 6 | 26 | 10 | 12 | 12.1 | 10.706 | -85.084 | 5.2 | 14 | 0.2 | 2.4 |
| 2011 | 6 | 26 | 10 | 20 | 13.7 | 10.703 | -85.084 | 5.0 | 17 | 0.2 | 2.5 |
| 2011 | 7 | 12 | 20 | 11 | 0.8 | 10.775 | -85.088 | 3.4 | 26 | 0.1 | 4.3 |
| 2011 | 7 | 12 | 20 | 28 | 13.6 | 10.734 | -85.105 | 10.6 | 19 | 0.3 | 2.8 |
| 2011 | 7 | 12 | 21 | 5 | 42.5 | 10.749 | -85.124 | 4.2 | 20 | 0.2 | 2.8 |
| 2011 | 7 | 12 | 21 | 35 | 20.0 | 10.737 | -85.128 | 4.2 | 16 | 0.3 | 2.2 |
| 2011 | 7 | 12 | 22 | 8 | 20.3 | 10.748 | -85.122 | 4.2 | 19 | 0.2 | 2.9 |
| 2011 | 7 | 12 | 22 | 42 | 23.8 | 10.707 | -85.105 | 10.8 | 12 | 0.2 | 2.0 |
| 2011 | 7 | 12 | 22 | 56 | 49.0 | 10.742 | -85.127 | 4.3 | 18 | 0.2 | 2.1 |
| 2011 | 7 | 12 | 23 | 13 | 53.3 | 10.740 | -85.121 | 4.0 | 11 | 0.1 | 1.7 |
| 2011 | 7 | 12 | 23 | 20 | 51.2 | 10.744 | -85.122 | 4.5 | 17 | 0.2 | 2.9 |
| 2011 | 7 | 12 | 23 | 30 | 12.5 | 10.748 | -85.122 | 8.5 | 16 | 0.2 | 1.7 |
| 2011 | 7 | 12 | 23 | 32 | 27.9 | 10.709 | -85.130 | 6.7 | 12 | 0.2 | 2.3 |
| 2011 | 7 | 13 | 0 | 26 | 18.1 | 10.744 | -85.126 | 8.1 | 12 | 0.1 | 2.8 |
| 2011 | 7 | 13 | 1 | 9 | 17.9 | 10.727 | -85.107 | 4.1 | 19 | 0.2 | 2.7 |
| 2011 | 7 | 13 | 1 | 59 | 40.6 | 10.725 | -85.096 | 4.2 | 9 | 0.2 | 2.1 |
| 2011 | 7 | 13 | 13 | 42 | 52.7 | 10.715 | -85.090 | 6.4 | 8 | 0.2 | 2.4 |
| 2011 | 7 | 13 | 14 | 53 | 31.0 | 10.746 | -85.128 | 5.0 | 16 | 0.1 | 3.1 |
| 2011 | 7 | 13 | 16 | 44 | 28.9 | 10.743 | -85.122 | 5.0 | 16 | 0.2 | 2.7 |
| 2011 | 7 | 14 | 0 | 19 | 37.4 | 10.745 | -85.130 | 5.4 | 12 | 0.2 | 2.8 |
| 2011 | 7 | 14 | 9 | 16 | 14.0 | 10.741 | -85.145 | 8.1 | 11 | 0.2 | 2.2 |
| 2011 | 7 | 14 | 9 | 31 | 1.8 | 10.693 | -85.092 | 7.1 | 18 | 0.1 | 2.9 |
| 2011 | 7 | 14 | 10 | 10 | 39.0 | 10.738 | -85.119 | 4.3 | 13 | 0.2 | 2.6 |
| 2011 | 7 | 14 | 17 | 8 | 9.2 | 10.737 | -85.130 | 4.7 | 12 | 0.1 | 2.4 |
| 2011 | 7 | 15 | 19 | 50 | 2.0 | 10.706 | -85.076 | 8.0 | 11 | 0.1 | 2.6 |
| 2011 | 7 | 16 | 2 | 19 | 32.7 | 10.704 | -85.074 | 5.6 | 8 | 0.1 | 2.4 |
| 2011 | 7 | 16 | 15 | 11 | 12.1 | 10.701 | -85.086 | 5.0 | 10 | 0.2 | 2.2 |
| 2011 | 7 | 17 | 15 | 51 | 35.8 | 10.693 | -85.089 | 5.0 | 9 | 0.1 | 2.2 |
| 2011 | 7 | 17 | 16 | 3 | 53.0 | 10.700 | -85.090 | 4.4 | 9 | 0.1 | 2.1 |
| 2011 | 7 | 18 | 22 | 46 | 56.4 | 10.700 | -85.087 | 8.6 | 8 | 0.2 | 2.1 |
| 2011 | 7 | 18 | 22 | 47 | 22.7 | 10.714 | -85.080 | 6.7 | 8 | 0.1 | 2.3 |
| 2011 | 7 | 18 | 22 | 55 | 29.5 | 10.725 | -85.105 | 4.2 | 9 | 0.2 | 2.2 |
| 2011 | 7 | 19 | 23 | 55 | 34.6 | 10.726 | -85.095 | 4.4 | 9 | 0.1 | 2.1 |
| 2011 | 7 | 20 | 9 | 19 | 55.4 | 10.710 | -85.084 | 5.2 | 9 | 0.2 | 2.5 |
| 2011 | 7 | 20 | 13 | 43 | 39.3 | 10.726 | -85.107 | 4.0 | 11 | 0.2 | 2.4 |
| 2011 | 7 | 21 | 4 | 7 | 11.6 | 10.708 | -85.083 | 5.2 | 12 | 0.2 | 2.6 |
| 2011 | 7 | 21 | 4 | 29 | 57.4 | 10.733 | -85.099 | 4.1 | 12 | 0.2 | 2.5 |
| 2011 | 7 | 29 | 8 | 25 | 3.8 | 10.605 | -84.977 | 4.9 | 19 | 0.2 | 2.8 |
| 2011 | 8 | 1 | 5 | 44 | 8.9 | 10.728 | -85.108 | 3.8 | 9 | 0.1 | 2.6 |
| 2011 | 8 | 1 | 8 | 10 | 44.5 | 10.742 | -85.121 | 4.3 | 11 | 0.2 | 2.6 |
| 2011 | 9 | 5 | 6 | 21 | 6.9 | 10.714 | -85.110 | 4.5 | 10 | 0.1 | 2.7 |
| 2011 | 10 | 1 | 17 | 58 | 27.2 | 10.764 | -85.129 | 7.6 | 8 | 0.1 | 2.5 |
| 2011 | 10 | 2 | 13 | 0 | 16.3 | 10.768 | -85.135 | 4.7 | 18 | 0.1 | 3.6 |
| 2011 | 10 | 2 | 13 | 59 | 30.0 | 10.757 | -85.088 | 5.1 | 12 | 0.1 | 3.0 |
| 2011 | 10 | 2 | 14 | 4 | 24.2 | 10.759 | -85.090 | 5.1 | 12 | 0.1 | 2.8 |
| 2011 | 10 | 2 | 20 | 32 | 3.9 | 10.752 | -85.083 | 6.4 | 11 | 0.1 | 3.0 |
| 2011 | 10 | 5 | 15 | 20 | 12.9 | 10.746 | -85.101 | 5.0 | 8 | 0.1 | 2.8 |
| 2011 | 10 | 22 | 1 | 48 | 45.8 | 10.766 | -85.117 | 5.2 | 13 | 0.1 | 2.8 |
| 2011 | 11 | 10 | 0 | 51 | 59.5 | 10.763 | -85.131 | 3.9 | 12 | 0.2 | 2.6 |
| 2011 | 11 | 18 | 23 | 46 | 56.4 | 10.764 | -85.119 | 7.7 | 18 | 0.1 | 3.0 |
| 2011 | 11 | 19 | 0 | 11 | 47.4 | 10.761 | -85.122 | 5.5 | 9 | 0.1 | 2.7 |
| 2011 | 11 | 24 | 10 | 32 | 20.4 | 10.772 | -85.127 | 8.4 | 13 | 0.4 | 2.7 |
| 2011 | 11 | 27 | 5 | 8 | 28.8 | 10.757 | -85.112 | 4.6 | 15 | 0.2 | 2.7 |
| 2011 | 12 | 5 | 2 | 45 | 34.3 | 10.709 | -85.103 | 4.7 | 9 | 0.1 | 2.1 |
| 2012 | 1 | 12 | 6 | 42 | 55.6 | 10.769 | -85.125 | 6.0 | 16 | 0.2 | 2.7 |
| 2012 | 1 | 23 | 10 | 56 | 26.3 | 10.680 | -85.092 | 7.4 | 16 | 0.1 | 2.5 |
| 2012 | 2 | 1 | 16 | 26 | 17.2 | 10.764 | -85.117 | 6.2 | 11 | 0.1 | 2.6 |
| 2012 | 2 | 2 | 2 | 0 | 39.3 | 10.619 | -84.988 | 7.1 | 19 | 0.2 | 3.0 |
| 2012 | 2 | 2 | 9 | 57 | 39.7 | 10.764 | -85.106 | 5.0 | 11 | 0.2 | 2.8 |
| 2012 | 2 | 14 | 4 | 21 | 51.2 | 10.759 | -85.119 | 7.8 | 9 | 0.2 | 2.7 |
| 2012 | 2 | 21 | 11 | 3 | 18.0 | 10.608 | -84.960 | 5.0 | 14 | 0.2 | 3.0 |
| 2012 | 2 | 28 | 0 | 29 | 17.3 | 10.649 | -85.030 | 2.5 | 9 | 0.2 | 2.8 |
| 2012 | 4 | 1 | 13 | 49 | 2.7 | 10.671 | -85.018 | 4.2 | 19 | 0.1 | 3.3 |
| 2012 | 4 | 13 | 23 | 35 | 17.4 | 10.718 | -85.096 | 5.0 | 12 | 0.1 | 2.6 |
| 2012 | 4 | 22 | 2 | 9 | 59.2 | 10.601 | -84.948 | 7.1 | 13 | 0.2 | 2.5 |
| 2012 | 5 | 2 | 1 | 33 | 48.3 | 10.564 | -84.948 | 4.6 | 15 | 0.2 | 2.6 |
| 2012 | 5 | 23 | 4 | 46 | 56.6 | 10.623 | -84.975 | 4.4 | 8 | 0.2 | 2.8 |
| 2012 | 5 | 23 | 9 | 7 | 41.4 | 10.615 | -84.981 | 4.7 | 18 | 0.2 | 3.2 |
| 2012 | 5 | 30 | 8 | 43 | 34.3 | 10.734 | -85.055 | 4.7 | 16 | 0.1 | 3.1 |
| 2012 | 7 | 26 | 3 | 58 | 17.2 | 10.747 | -85.074 | 5.0 | 16 | 0.1 | 2.8 |
| 2012 | 7 | 26 | 4 | 46 | 17.4 | 10.745 | -85.076 | 3.7 | 20 | 0.2 | 3.0 |
| 2012 | 9 | 5 | 17 | 57 | 56.4 | 10.611 | -84.945 | 4.1 | 10 | 0.2 | 2.9 |
| 2012 | 9 | 6 | 6 | 36 | 24.0 | 10.651 | -85.055 | 3.4 | 9 | 0.1 | 2.7 |
| 2012 | 9 | 6 | 10 | 47 | 34.3 | 10.606 | -84.942 | 4.2 | 11 | 0.2 | 3.0 |
| 2012 | 9 | 7 | 0 | 55 | 0.8 | 10.650 | -85.056 | 4.1 | 11 | 0.1 | 2.8 |
| 2012 | 9 | 7 | 5 | 2 | 36.8 | 10.608 | -84.982 | 6.5 | 9 | 0.1 | 2.1 |
| 2012 | 9 | 17 | 1 | 11 | 8.6 | 10.638 | -84.996 | 7.1 | 20 | 0.2 | 3.1 |
| 2012 | 9 | 17 | 6 | 58 | 0.0 | 10.650 | -85.042 | 5.0 | 15 | 0.2 | 2.8 |
| 2012 | 9 | 21 | 6 | 6 | 42.3 | 10.641 | -84.972 | 4.6 | 9 | 0.1 | 2.9 |
| 2012 | 9 | 22 | 0 | 15 | 54.2 | 10.644 | -85.032 | 3.8 | 14 | 0.1 | 2.9 |
| 2012 | 9 | 23 | 14 | 49 | 35.3 | 10.663 | -84.997 | 4.5 | 28 | 0.2 | 3.2 |
| 2012 | 9 | 23 | 19 | 26 | 31.8 | 10.660 | -84.992 | 4.3 | 11 | 0.1 | 2.8 |
| 2012 | 9 | 24 | 13 | 9 | 10.1 | 10.642 | -84.973 | 4.4 | 8 | 0.1 | 2.5 |
| 2012 | 9 | 27 | 0 | 23 | 30.6 | 10.592 | -84.912 | 4.5 | 13 | 0.2 | 2.4 |
| 2012 | 9 | 27 | 16 | 59 | 30.1 | 10.649 | -85.027 | 4.0 | 14 | 0.1 | 2.6 |
| 2012 | 9 | 27 | 18 | 19 | 0.9 | 10.654 | -84.989 | 4.5 | 10 | 0.1 | 2.8 |
| 2012 | 9 | 30 | 3 | 23 | 10.6 | 10.656 | -84.990 | 4.4 | 10 | 0.1 | 2.3 |
| 2012 | 10 | 1 | 18 | 57 | 1.2 | 10.755 | -85.121 | 6.2 | 20 | 0.2 | 3.0 |
| 2012 | 10 | 1 | 23 | 36 | 22.8 | 10.683 | -85.011 | 4.7 | 9 | 0.1 | 2.5 |
| 2012 | 10 | 2 | 7 | 30 | 2.5 | 10.655 | -84.987 | 4.3 | 10 | 0.2 | 2.3 |
| 2012 | 10 | 5 | 15 | 53 | 45.9 | 10.657 | -84.988 | 4.8 | 9 | 0.1 | 2.4 |
| 2012 | 10 | 6 | 4 | 28 | 40.4 | 10.700 | -85.085 | 4.7 | 14 | 0.2 | 2.4 |
| 2012 | 10 | 7 | 12 | 17 | 56.6 | 10.609 | -84.989 | 5.1 | 10 | 0.1 | 1.9 |
| 2012 | 10 | 9 | 19 | 7 | 54.5 | 10.641 | -85.010 | 4.3 | 10 | 0.3 | 2.4 |
| 2012 | 10 | 11 | 17 | 6 | 49.8 | 10.644 | -85.022 | 3.5 | 27 | 0.2 | 3.2 |
| 2012 | 10 | 16 | 20 | 44 | 47.9 | 10.584 | -84.988 | 7.4 | 10 | 0.2 | 2.2 |
| 2012 | 10 | 19 | 1 | 3 | 17.7 | 10.569 | -84.934 | 3.6 | 15 | 0.1 | 2.7 |
| 2012 | 10 | 20 | 20 | 35 | 11.6 | 10.651 | -85.051 | 4.3 | 11 | 0.2 | 2.3 |
| 2012 | 10 | 21 | 4 | 44 | 52.5 | 10.595 | -84.949 | 9.1 | 36 | 0.1 | 3.2 |
| 2012 | 10 | 21 | 4 | 53 | 27.4 | 10.597 | -84.951 | 8.2 | 17 | 0.2 | 2.7 |
| 2012 | 10 | 21 | 5 | 2 | 40.4 | 10.599 | -84.945 | 9.0 | 26 | 0.2 | 2.9 |
| 2012 | 10 | 21 | 6 | 28 | 15.3 | 10.598 | -84.949 | 7.5 | 15 | 0.2 | 2.2 |
| 2012 | 10 | 22 | 14 | 44 | 12.2 | 10.598 | -84.971 | 7.9 | 17 | 0.1 | 2.2 |
| 2012 | 10 | 22 | 15 | 18 | 40.7 | 10.603 | -84.963 | 7.1 | 9 | 0.1 | 1.9 |
| 2012 | 10 | 23 | 0 | 47 | 48.6 | 10.601 | -84.974 | 7.0 | 12 | 0.1 | 2.3 |
| 2012 | 10 | 28 | 6 | 34 | 10.6 | 10.619 | -84.992 | 5.8 | 12 | 0.1 | 2.4 |
| 2012 | 10 | 30 | 21 | 42 | 18.5 | 10.610 | -84.940 | 4.3 | 14 | 0.2 | 2.7 |
| 2012 | 11 | 2 | 3 | 45 | 34.2 | 10.663 | -84.981 | 3.2 | 10 | 0.2 | 2.7 |
| 2012 | 11 | 3 | 14 | 0 | 17.6 | 10.660 | -84.980 | 4.5 | 10 | 0.2 | 2.6 |
| 2012 | 11 | 4 | 0 | 37 | 9.8 | 10.658 | -84.990 | 4.1 | 31 | 0.2 | 3.4 |
| 2012 | 11 | 5 | 1 | 22 | 29.9 | 10.671 | -85.003 | 4.4 | 14 | 0.2 | 2.5 |
| 2012 | 11 | 6 | 14 | 11 | 19.6 | 10.654 | -85.080 | 4.9 | 9 | 0.3 | 2.5 |
| 2012 | 11 | 7 | 8 | 37 | 50.2 | 10.603 | -84.953 | 6.0 | 11 | 0.1 | 2.2 |
| 2012 | 11 | 23 | 0 | 59 | 30.2 | 10.625 | -84.960 | 4.2 | 20 | 0.2 | 3.0 |
| 2012 | 11 | 26 | 23 | 20 | 30.9 | 10.575 | -84.928 | 8.6 | 17 | 0.2 | 2.6 |
| 2012 | 11 | 30 | 0 | 45 | 6.9 | 10.660 | -84.992 | 4.3 | 10 | 0.2 | 2.2 |
| 2012 | 12 | 20 | 17 | 30 | 5.2 | 10.672 | -85.008 | 5.9 | 36 | 0.2 | 3.3 |
| 2012 | 12 | 24 | 9 | 3 | 37.5 | 10.501 | -84.935 | 5.0 | 19 | 0.3 | 3.0 |
| 2012 | 12 | 24 | 9 | 6 | 12.3 | 10.500 | -84.935 | 5.0 | 20 | 0.3 | 3.0 |
| 2013 | 1 | 21 | 8 | 15 | 32.9 | 10.662 | -85.022 | 4.7 | 14 | 0.3 | 2.9 |
| 2013 | 2 | 27 | 7 | 57 | 4.3 | 10.598 | -84.952 | 9.4 | 8 | 0.1 | 2.4 |
| 2013 | 4 | 27 | 6 | 37 | 30.4 | 10.592 | -84.991 | 8.3 | 20 | 0.2 | 2.8 |
| 2013 | 5 | 15 | 23 | 7 | 7.2 | 10.538 | -84.851 | 5.0 | 10 | 0.3 | 2.5 |
| 2013 | 6 | 2 | 23 | 25 | 53.5 | 10.768 | -85.103 | 6.0 | 11 | 0.2 | 2.8 |
| 2013 | 6 | 3 | 3 | 0 | 56.9 | 10.758 | -85.118 | 5.0 | 23 | 0.3 | 2.9 |
| 2013 | 6 | 5 | 4 | 46 | 41.4 | 10.761 | -85.083 | 5.0 | 10 | 0.2 | 2.9 |
| 2013 | 6 | 7 | 1 | 3 | 31.9 | 10.734 | -85.143 | 3.2 | 10 | 0.2 | 2.6 |
| 2013 | 6 | 21 | 22 | 17 | 51.1 | 10.608 | -84.961 | 7.5 | 18 | 0.1 | 2.7 |
| 2013 | 7 | 10 | 1 | 44 | 11.9 | 10.568 | -84.927 | 5.0 | 20 | 0.3 | 2.9 |
| 2013 | 7 | 12 | 15 | 29 | 6.3 | 10.579 | -84.924 | 5.6 | 23 | 0.2 | 2.8 |
| 2013 | 7 | 21 | 3 | 44 | 25.9 | 10.759 | -85.085 | 6.4 | 13 | 0.1 | 2.9 |
| 2013 | 8 | 20 | 20 | 17 | 30.2 | 10.620 | -84.967 | 6.3 | 28 | 0.2 | 3.2 |
| 2013 | 8 | 23 | 4 | 56 | 29.3 | 10.609 | -84.964 | 5.3 | 23 | 0.2 | 3.0 |
| 2013 | 8 | 29 | 6 | 57 | 18.9 | 10.631 | -84.972 | 5.8 | 23 | 0.2 | 3.0 |
| 2013 | 8 | 29 | 9 | 9 | 57.0 | 10.703 | -85.071 | 4.8 | 24 | 0.2 | 3.1 |
| 2013 | 9 | 1 | 3 | 9 | 32.0 | 10.668 | -84.993 | 3.9 | 12 | 0.2 | 2.8 |
| 2013 | 9 | 6 | 6 | 25 | 26.5 | 10.730 | -85.037 | 4.8 | 21 | 0.2 | 3.1 |
| 2013 | 9 | 7 | 9 | 42 | 43.8 | 10.726 | -85.033 | 4.9 | 9 | 0.2 | 3.0 |
| 2013 | 9 | 8 | 7 | 33 | 1.7 | 10.690 | -85.017 | 5.0 | 13 | 0.2 | 3.0 |
| 2013 | 9 | 8 | 7 | 35 | 52.5 | 10.690 | -85.016 | 5.0 | 9 | 0.2 | 2.7 |
| 2013 | 9 | 13 | 3 | 31 | 21.6 | 10.663 | -85.004 | 4.7 | 27 | 0.2 | 2.9 |
| 2013 | 9 | 15 | 2 | 25 | 55.2 | 10.700 | -85.069 | 4.9 | 9 | 0.1 | 2.6 |
| 2013 | 9 | 16 | 5 | 17 | 59.0 | 10.669 | -85.026 | 3.3 | 8 | 0.1 | 2.7 |
| 2013 | 9 | 28 | 1 | 41 | 37.5 | 10.614 | -84.960 | 5.8 | 28 | 0.2 | 3.2 |
| 2013 | 10 | 4 | 0 | 22 | 17.0 | 10.620 | -84.974 | 4.6 | 14 | 0.1 | 2.8 |
| 2013 | 10 | 5 | 0 | 36 | 25.3 | 10.626 | -84.976 | 4.3 | 25 | 0.2 | 2.9 |
| 2013 | 10 | 5 | 4 | 21 | 49.2 | 10.621 | -84.972 | 4.3 | 10 | 0.3 | 2.8 |
| 2013 | 10 | 21 | 11 | 49 | 51.0 | 10.842 | -85.141 | 5.0 | 19 | 0.2 | 3.1 |
| 2013 | 11 | 10 | 19 | 4 | 4.8 | 10.777 | -85.100 | 7.0 | 14 | 0.3 | 2.7 |
| 2013 | 11 | 17 | 15 | 13 | 51.6 | 10.686 | -85.096 | 26.5 | 18 | 0.5 | 3.1 |
| 2013 | 11 | 21 | 1 | 17 | 6.0 | 10.668 | -85.004 | 4.2 | 14 | 0.2 | 3.0 |
| 2014 | 1 | 11 | 9 | 31 | 38.9 | 10.810 | -85.128 | 2.8 | 8 | 0.2 | 2.8 |
| 2014 | 1 | 21 | 8 | 41 | 27.3 | 10.517 | -84.955 | 5.7 | 8 | 0.1 | 2.4 |
| 2014 | 1 | 21 | 12 | 29 | 9.9 | 10.712 | -85.023 | 4.3 | 10 | 0.1 | 2.7 |
| 2014 | 1 | 23 | 23 | 36 | 53.5 | 10.519 | -84.953 | 5.3 | 11 | 0.1 | 2.7 |
| 2014 | 2 | 3 | 7 | 32 | 34.6 | 10.678 | -85.009 | 4.9 | 11 | 0.2 | 2.9 |
| 2014 | 2 | 4 | 1 | 33 | 15.8 | 10.631 | -84.975 | 7.2 | 9 | 0.1 | 2.8 |
| 2014 | 2 | 27 | 7 | 24 | 20.4 | 10.619 | -84.967 | 4.7 | 15 | 0.1 | 3.1 |
| 2014 | 3 | 11 | 3 | 25 | 18.8 | 10.644 | -85.042 | 4.3 | 8 | 0.1 | 2.6 |
| 2014 | 3 | 11 | 5 | 39 | 48.1 | 10.656 | -85.042 | 5.0 | 11 | 0.2 | 2.9 |
| 2014 | 3 | 11 | 8 | 12 | 18.2 | 10.652 | -85.043 | 4.2 | 10 | 0.1 | 2.9 |
| 2014 | 3 | 11 | 21 | 25 | 1.8 | 10.709 | -85.018 | 5.0 | 8 | 0.2 | 3.0 |
| 2014 | 3 | 12 | 15 | 1 | 34.4 | 10.798 | -85.128 | 4.4 | 21 | 0.2 | 3.2 |
| 2014 | 3 | 12 | 19 | 44 | 29.9 | 10.757 | -85.092 | 5.0 | 16 | 0.3 | 3.2 |
| 2014 | 3 | 26 | 1 | 46 | 5.2 | 10.766 | -85.089 | 7.5 | 10 | 0.2 | 2.9 |
| 2014 | 3 | 27 | 18 | 10 | 22.0 | 10.766 | -85.116 | 5.0 | 13 | 0.2 | 3.1 |
| 2014 | 4 | 7 | 6 | 48 | 34.4 | 10.632 | -84.971 | 4.8 | 8 | 0.2 | 2.5 |
| 2014 | 4 | 10 | 19 | 7 | 36.1 | 10.589 | -84.917 | 6.5 | 11 | 0.2 | 3.1 |
| 2014 | 4 | 10 | 19 | 16 | 13.4 | 10.582 | -84.923 | 5.4 | 11 | 0.2 | 2.9 |
| 2014 | 4 | 28 | 11 | 8 | 13.0 | 10.626 | -84.971 | 4.6 | 8 | 0.1 | 3.0 |
| 2014 | 5 | 12 | 17 | 22 | 48.6 | 10.599 | -84.932 | 5.1 | 12 | 0.2 | 3.1 |
| 2014 | 5 | 13 | 3 | 18 | 27.0 | 10.753 | -85.065 | 3.4 | 8 | 0.3 | 3.1 |
| 2014 | 5 | 25 | 12 | 1 | 13.2 | 10.664 | -84.993 | 4.8 | 10 | 0.1 | 3.0 |
| 2014 | 5 | 29 | 10 | 20 | 8.4 | 10.695 | -85.096 | 4.6 | 18 | 0.2 | 2.8 |
| 2014 | 5 | 30 | 3 | 22 | 58.9 | 10.615 | -84.960 | 4.5 | 8 | 0.1 | 2.5 |
| 2014 | 5 | 30 | 3 | 24 | 27.1 | 10.618 | -84.959 | 4.4 | 8 | 0.1 | 2.5 |
| 2014 | 6 | 4 | 21 | 37 | 21.9 | 10.613 | -84.956 | 8.5 | 14 | 0.3 | 3.0 |
| 2014 | 6 | 13 | 1 | 59 | 30.5 | 10.783 | -85.117 | 4.1 | 8 | 0.1 | 2.5 |
| 2014 | 6 | 30 | 15 | 35 | 19.0 | 10.764 | -85.109 | 6.0 | 12 | 0.2 | 3.0 |
| 2014 | 7 | 12 | 21 | 44 | 43.3 | 10.793 | -85.121 | 4.9 | 9 | 0.1 | 3.1 |
| 2014 | 7 | 13 | 17 | 18 | 35.5 | 10.756 | -85.074 | 5.0 | 9 | 0.2 | 3.1 |
| 2014 | 7 | 16 | 11 | 43 | 31.1 | 10.676 | -85.011 | 5.2 | 11 | 0.1 | 3.0 |
| 2014 | 7 | 19 | 7 | 10 | 12.4 | 10.812 | -85.129 | 5.1 | 8 | 0.1 | 2.9 |
| 2014 | 7 | 24 | 11 | 41 | 17.3 | 10.793 | -85.124 | 2.5 | 16 | 0.3 | 3.0 |
| 2014 | 8 | 2 | 20 | 17 | 16.3 | 10.638 | -84.993 | 3.8 | 13 | 0.5 | 2.6 |
| 2014 | 8 | 2 | 23 | 47 | 27.6 | 10.637 | -84.979 | 4.4 | 8 | 0.1 | 2.3 |
| 2014 | 8 | 3 | 0 | 8 | 6.2 | 10.635 | -84.974 | 4.8 | 9 | 0.2 | 2.1 |
| 2014 | 8 | 3 | 2 | 51 | 22.2 | 10.635 | -84.983 | 6.0 | 8 | 0.1 | 2.0 |
| 2014 | 8 | 3 | 3 | 31 | 52.5 | 10.633 | -84.986 | 6.9 | 19 | 0.2 | 2.7 |
| 2014 | 8 | 3 | 4 | 4 | 32.4 | 10.639 | -84.979 | 4.2 | 9 | 0.2 | 2.2 |
| 2014 | 8 | 3 | 4 | 22 | 25.2 | 10.635 | -84.984 | 3.9 | 16 | 0.5 | 2.2 |
| 2014 | 8 | 3 | 4 | 27 | 57.6 | 10.636 | -84.979 | 4.6 | 8 | 0.1 | 2.4 |
| 2014 | 8 | 3 | 5 | 49 | 11.6 | 10.631 | -84.993 | 4.5 | 37 | 0.5 | 3.3 |
| 2014 | 8 | 3 | 6 | 19 | 40.5 | 10.631 | -84.980 | 8.2 | 9 | 0.3 | 2.4 |
| 2014 | 8 | 8 | 23 | 7 | 11.5 | 10.717 | -85.030 | 8.5 | 10 | 0.2 | 2.1 |
| 2014 | 8 | 25 | 5 | 52 | 11.6 | 10.641 | -84.986 | 4.4 | 8 | 0.1 | 2.1 |
| 2014 | 9 | 1 | 15 | 33 | 4.9 | 10.688 | -85.012 | 5.8 | 9 | 0.1 | 2.3 |
| 2014 | 9 | 9 | 7 | 40 | 31.2 | 10.621 | -84.950 | 6.8 | 9 | 0.2 | 1.9 |
| 2014 | 9 | 16 | 1 | 10 | 5.9 | 10.552 | -84.806 | 8.4 | 15 | 0.2 | 2.5 |
| 2014 | 9 | 17 | 21 | 27 | 52.5 | 10.681 | -85.010 | 4.9 | 10 | 0.1 | 2.6 |
| 2014 | 10 | 7 | 7 | 29 | 29.2 | 10.656 | -84.992 | 4.0 | 9 | 0.1 | 2.4 |
| 2014 | 10 | 15 | 3 | 46 | 30.2 | 10.683 | -85.013 | 5.0 | 8 | 0.1 | 1.7 |
| 2014 | 10 | 15 | 3 | 46 | 50.6 | 10.682 | -85.019 | 6.7 | 19 | 0.2 | 2.2 |
| 2014 | 10 | 16 | 19 | 32 | 12.0 | 10.592 | -84.939 | 10.7 | 24 | 0.1 | 3.5 |
| 2014 | 11 | 17 | 16 | 7 | 21.3 | 10.651 | -85.009 | 4.6 | 15 | 0.2 | 2.6 |
| 2014 | 12 | 1 | 11 | 40 | 43.9 | 10.601 | -84.967 | 3.9 | 11 | 0.3 | 2.4 |
| 2014 | 12 | 3 | 17 | 4 | 19.4 | 10.716 | -85.026 | 4.9 | 13 | 0.2 | 2.8 |
| 2014 | 12 | 22 | 8 | 53 | 16.1 | 10.601 | -84.947 | 7.8 | 11 | 0.2 | 1.9 |
| 2014 | 12 | 30 | 15 | 18 | 28.9 | 10.620 | -84.973 | 5.8 | 26 | 0.3 | 2.7 |
| 2014 | 12 | 31 | 5 | 14 | 7.2 | 10.623 | -84.966 | 4.9 | 21 | 0.3 | 2.4 |
| 2015 | 1 | 7 | 4 | 33 | 29.7 | 10.622 | -84.971 | 8.6 | 8 | 0.2 | 2.4 |
| 2015 | 1 | 8 | 20 | 26 | 40.5 | 10.575 | -84.878 | 8.0 | 15 | 0.2 | 2.8 |
| 2015 | 1 | 8 | 22 | 12 | 27.1 | 10.589 | -84.881 | 7.2 | 11 | 0.2 | 2.4 |
| 2015 | 1 | 17 | 23 | 1 | 46.4 | 10.766 | -85.097 | 4.9 | 9 | 0.1 | 2.6 |
| 2015 | 2 | 15 | 7 | 55 | 53.9 | 10.698 | -85.025 | 2.5 | 40 | 0.4 | 3.4 |
| 2015 | 3 | 8 | 4 | 28 | 14.1 | 10.580 | -84.944 | 8.7 | 15 | 0.4 | 2.6 |
| 2015 | 3 | 17 | 2 | 43 | 38.0 | 10.579 | -84.929 | 57.0 | 31 | 0.3 | 2.8 |
| 2015 | 3 | 23 | 1 | 50 | 22.9 | 10.774 | -85.113 | 5.2 | 11 | 0.2 | 2.8 |
| 2015 | 5 | 3 | 0 | 45 | 14.8 | 10.632 | -84.992 | 5.0 | 28 | 0.5 | 2.4 |
| 2015 | 5 | 6 | 2 | 59 | 51.0 | 10.576 | -84.924 | 6.0 | 13 | 0.2 | 2.0 |
| 2015 | 5 | 23 | 19 | 54 | 55.4 | 10.692 | -85.092 | 5.0 | 8 | 0.3 | 2.3 |
| 2015 | 6 | 1 | 16 | 28 | 53.3 | 10.668 | -84.986 | 3.8 | 10 | 0.2 | 2.4 |
| 2015 | 6 | 4 | 18 | 36 | 29.4 | 10.830 | -85.190 | 5.0 | 16 | 0.5 | 3.0 |
| 2015 | 7 | 5 | 2 | 24 | 17.4 | 10.587 | -84.941 | 8.4 | 28 | 0.3 | 2.8 |
| 2015 | 7 | 5 | 2 | 24 | 17.4 | 10.596 | -84.943 | 8.7 | 21 | 0.2 | 3.1 |
| 2015 | 7 | 13 | 11 | 56 | 6.3 | 10.656 | -85.001 | 5.0 | 11 | 0.2 | 2.8 |
| 2015 | 7 | 19 | 5 | 32 | 14.2 | 10.738 | -85.106 | 4.9 | 12 | 0.2 | 2.6 |
| 2015 | 7 | 22 | 9 | 8 | 50.2 | 10.754 | -85.071 | 5.0 | 19 | 0.1 | 2.8 |
| 2015 | 7 | 30 | 3 | 4 | 27.3 | 10.610 | -84.929 | 4.5 | 10 | 0.1 | 2.6 |
| 2015 | 8 | 2 | 17 | 51 | 0.3 | 10.587 | -84.909 | 4.6 | 29 | 0.8 | 2.5 |
| 2015 | 9 | 16 | 18 | 42 | 32.0 | 10.652 | -84.987 | 4.9 | 8 | 0.1 | 2.8 |
| 2015 | 10 | 18 | 4 | 7 | 45.6 | 10.612 | -84.992 | 5.9 | 21 | 0.2 | 2.6 |
| 2015 | 10 | 18 | 13 | 4 | 6.6 | 10.614 | -84.992 | 6.7 | 10 | 0.1 | 2.3 |
| 2015 | 10 | 27 | 10 | 33 | 59.2 | 10.636 | -84.966 | 7.8 | 49 | 0.3 | 3.2 |
| 2015 | 11 | 11 | 4 | 13 | 11.4 | 10.716 | -85.061 | 6.0 | 10 | 0.1 | 2.2 |
| 2015 | 11 | 13 | 0 | 47 | 10.3 | 10.713 | -85.027 | 5.7 | 10 | 0.2 | 2.2 |
| 2015 | 11 | 18 | 18 | 36 | 0.3 | 10.573 | -84.914 | 9.2 | 17 | 0.3 | 2.3 |
| 2015 | 11 | 27 | 10 | 16 | 9.9 | 10.731 | -85.096 | 7.2 | 8 | 0.1 | 2.4 |
| 2015 | 12 | 4 | 14 | 59 | 33.0 | 10.701 | -85.056 | 4.9 | 11 | 0.0 | 2.9 |
| 2015 | 12 | 4 | 16 | 3 | 59.5 | 10.702 | -85.057 | 4.9 | 8 | 0.1 | 2.5 |
| 2015 | 12 | 4 | 16 | 12 | 21.2 | 10.693 | -85.065 | 5.0 | 63 | 0.3 | 3.6 |
| 2015 | 12 | 4 | 23 | 34 | 49.7 | 10.688 | -85.057 | 5.0 | 28 | 0.4 | 3.1 |
| 2015 | 12 | 21 | 16 | 25 | 5.3 | 10.665 | -84.998 | 5.0 | 27 | 0.3 | 2.9 |
| 2015 | 12 | 25 | 12 | 49 | 35.8 | 10.775 | -85.084 | 7.3 | 8 | 0.1 | 2.9 |
| 2015 | 12 | 30 | 16 | 2 | 47.8 | 10.769 | -85.077 | 6.7 | 16 | 0.1 | 3.3 |
| 2016 | 1 | 5 | 9 | 6 | 43.5 | 10.826 | -85.145 | 4.7 | 12 | 0.2 | 2.7 |
| 2016 | 1 | 11 | 17 | 28 | 27.2 | 10.767 | -85.088 | 6.1 | 9 | 0.2 | 2.5 |
| 2016 | 1 | 13 | 20 | 52 | 25.2 | 10.745 | -85.076 | 6.9 | 15 | 0.3 | 2.3 |
| 2016 | 1 | 31 | 1 | 4 | 5.9 | 10.598 | -84.967 | 7.7 | 14 | 0.2 | 2.6 |
| 2016 | 2 | 16 | 8 | 5 | 16.9 | 10.665 | -84.995 | 4.8 | 8 | 0.1 | 2.5 |
| 2016 | 3 | 16 | 13 | 43 | 22.6 | 10.696 | -85.009 | 4.9 | 8 | 0.1 | 2.1 |
| 2016 | 3 | 26 | 5 | 15 | 31.7 | 10.625 | -84.981 | 1.2 | 13 | 0.8 | 2.6 |
| 2016 | 3 | 26 | 15 | 23 | 57.0 | 10.705 | -85.097 | 4.2 | 12 | 0.1 | 2.5 |
| 2016 | 4 | 1 | 2 | 0 | 54.5 | 10.706 | -85.020 | 5.0 | 11 | 0.1 | 2.4 |
| 2016 | 5 | 2 | 4 | 40 | 5.8 | 10.528 | -84.870 | 10.4 | 10 | 0.1 | 2.2 |
| 2016 | 5 | 10 | 10 | 44 | 25.6 | 10.628 | -84.963 | 4.5 | 8 | 0.1 | 2.5 |
| 2016 | 5 | 22 | 0 | 7 | 25.4 | 10.684 | -85.031 | 3.4 | 27 | 0.6 | 3.1 |
| 2016 | 5 | 28 | 6 | 46 | 29.7 | 10.616 | -84.953 | 6.5 | 13 | 0.2 | 2.4 |
| 2016 | 7 | 3 | 1 | 58 | 29.1 | 10.760 | -85.060 | 0.5 | 131 | 0.6 | 5.4 |
| 2016 | 7 | 3 | 2 | 3 | 14.5 | 10.754 | -85.060 | 9.0 | 30 | 0.5 | 4.0 |
| 2016 | 7 | 3 | 2 | 16 | 11.5 | 10.765 | -85.053 | 3.7 | 45 | 0.6 | 5.1 |
| 2016 | 7 | 3 | 2 | 43 | 30.5 | 10.743 | -85.074 | 4.0 | 16 | 0.2 | 2.7 |
| 2016 | 7 | 3 | 2 | 44 | 37.6 | 10.753 | -85.063 | 5.2 | 15 | 0.5 | 2.5 |
| 2016 | 7 | 3 | 2 | 57 | 30.3 | 10.755 | -85.071 | 6.0 | 9 | 0.2 | 2.9 |
| 2016 | 7 | 3 | 3 | 35 | 34.4 | 10.796 | -85.099 | 5.4 | 10 | 0.2 | 2.6 |
| 2016 | 7 | 3 | 4 | 27 | 33.3 | 10.703 | -85.031 | 3.3 | 12 | 0.5 | 3.6 |
| 2016 | 7 | 3 | 4 | 51 | 38.9 | 10.772 | -85.101 | 6.0 | 12 | 0.5 | 2.6 |
| 2016 | 7 | 3 | 5 | 38 | 55.7 | 10.793 | -85.098 | 4.3 | 34 | 0.5 | 3.9 |
| 2016 | 7 | 3 | 8 | 22 | 38.7 | 10.748 | -85.057 | 0.0 | 13 | 0.8 | 2.8 |
| 2016 | 7 | 5 | 4 | 48 | 11.5 | 10.753 | -85.093 | 3.3 | 23 | 0.3 | 3.0 |
| 2016 | 7 | 9 | 3 | 55 | 41.3 | 10.770 | -85.125 | 4.3 | 9 | 0.2 | 2.5 |
| 2016 | 7 | 11 | 6 | 24 | 4.4 | 10.754 | -85.089 | 3.9 | 12 | 0.4 | 3.3 |
| 2016 | 7 | 19 | 15 | 17 | 46.9 | 10.774 | -85.095 | 10.0 | 14 | 0.5 | 2.7 |
| 2016 | 7 | 20 | 8 | 6 | 45.4 | 10.720 | -85.047 | 3.7 | 18 | 0.6 | 3.8 |
| 2016 | 7 | 24 | 3 | 11 | 40.6 | 10.709 | -85.035 | 1.0 | 13 | 0.3 | 2.7 |
| 2016 | 7 | 29 | 4 | 21 | 2.7 | 10.742 | -85.063 | 7.9 | 16 | 0.3 | 2.2 |

Supplementary Table S2. Earthquakes relocated using HypoDD^1^ with focal mechanisms determined using Focmec^2^. The column headings are as follows:

Y = year, M = month, D = day, H = hours, m = minutes and s = seconds, in GMT. Mag = Moment magnitude (M_w_). Lat = latitude and Long = longitude, in decimal degrees both referenced to the WGS84 reference frame. Depth = depth in km relative to mean sea level. RMS = root mean square amplitude. FMS = focal mechanism given as **strike dip rake** for one nodal plane with strike as azimuthal degrees, dip in degrees from local horizontal, and rake of slip direction in degrees from strike direction measured into upper hemisphere (positive) or into lower hemisphere (negative) and representing the motion of the hangingwall block.

| **Supplementary Table S2** | | | | | | | | | | | |
| --- | --- | --- | --- | --- | --- | --- | --- | --- | --- | --- | --- |
| Y | M | D | H | m | s | Mag | Lat | Long | Depth | RMS | FMS |
| 2007 | 12 | 5 | 3 | 27 | 39.62 | 3.3 | 10.635265 | -85.044385 | 6.638 | 0.034 |  |
| 2008 | 3 | 5 | 13 | 23 | 6.59 | 3 | 10.402603 | -85.071281 | 8.969 | 0.066 |  |
| 2008 | 3 | 5 | 16 | 10 | 48.51 | 3.8 | 10.402498 | -85.0736 | 8.694 | 0.066 |  |
| 2008 | 4 | 30 | 6 | 20 | 34.43 | 4.2 | 10.602275 | -84.995475 | 4.866 | 0.035 |  |
| 2008 | 5 | 2 | 6 | 31 | 23.41 | 2.3 | 10.6164 | -84.970874 | 3.292 | 0.065 |  |
| 2009 | 1 | 11 | 23 | 41 | 34.16 | 2 | 10.660311 | -85.189689 | 2.945 | 0.081 |  |
| 2009 | 5 | 10 | 3 | 19 | 12.77 | 2.7 | 10.665746 | -85.095524 | 2.95 | 0.041 |  |
| 2009 | 5 | 24 | 12 | 31 | 26.75 | 2.9 | 10.727462 | -85.171419 | 2.153 | 0.068 |  |
| 2009 | 5 | 24 | 12 | 34 | 14.02 | 2.6 | 10.726477 | -85.171965 | 2.272 | 0.059 |  |
| 2009 | 8 | 11 | 5 | 9 | 50.79 | 3.4 | 10.547329 | -84.970972 | 6.068 | 0.048 |  |
| 2009 | 8 | 11 | 7 | 43 | 22.32 | 2.9 | 10.547037 | -84.973299 | 5.797 | 0.048 |  |
| 2009 | 8 | 16 | 17 | 51 | 28.6 | 3 | 10.621381 | -84.995744 | 4.859 | 0.08 |  |
| 2009 | 10 | 27 | 15 | 59 | 54.47 | 2.6 | 10.624193 | -84.968058 | 4.017 | 0.064 |  |
| 2009 | 11 | 10 | 8 | 30 | 2.56 | 2.8 | 10.693264 | -85.034115 | 6.183 | 0.039 |  |
| 2009 | 12 | 28 | 3 | 44 | 39.76 | 2.5 | 10.618949 | -84.961865 | 4.951 | 0.094 |  |
| 2010 | 3 | 28 | 14 | 23 | 43.61 | 3 | 10.676731 | -84.995378 | 4.424 | 0.062 |  |
| 2010 | 7 | 16 | 8 | 12 | 7.04 | 2.9 | 10.652027 | -84.964054 | 4.595 | 0.109 |  |
| 2010 | 7 | 25 | 1 | 50 | 17.27 | 2.2 | 10.602323 | -84.984513 | 7.968 | 0.035 |  |
| 2010 | 9 | 22 | 10 | 18 | 24.25 | 2.9 | 10.718332 | -85.176172 | 2.622 | 0.066 |  |
| 2010 | 10 | 21 | 22 | 45 | 50.92 | 3.1 | 10.725486 | -85.172396 | 2.732 | 0.051 |  |
| 2010 | 10 | 27 | 5 | 15 | 54.16 | 3.1 | 10.657483 | -84.986182 | 5.146 | 0.078 |  |
| 2010 | 11 | 11 | 5 | 2 | 36.27 | 2.7 | 10.699974 | -85.021875 | 6.413 | 0.046 |  |
| 2010 | 12 | 17 | 4 | 56 | 10.02 | 3 | 10.697426 | -85.078182 | 6.334 | 0.051 | 180.00 55.00 -89.00 |
| 2010 | 12 | 17 | 9 | 5 | 16.85 | 2.7 | 10.692581 | -85.07535 | 6.421 | 0.046 |  |
| 2010 | 12 | 17 | 9 | 8 | 35.82 | 2.2 | 10.694551 | -85.075439 | 6.039 | 0.031 |  |
| 2010 | 12 | 17 | 9 | 9 | 2.29 | 2.2 | 10.693468 | -85.075586 | 6.261 | 0.027 |  |
| 2010 | 12 | 18 | 17 | 53 | 0.78 | 2.7 | 10.694653 | -85.076204 | 6.464 | 0.043 |  |
| 2010 | 12 | 25 | 22 | 59 | 9.98 | 2.7 | 10.695807 | -85.075529 | 6.307 | 0.06 |  |
| 2011 | 1 | 16 | 5 | 8 | 10.26 | 2.3 | 10.705522 | -85.085384 | 5.263 | 0.046 |  |
| 2011 | 2 | 17 | 11 | 6 | 1.16 | 2.4 | 10.737107 | -85.173535 | 2.39 | 0.029 |  |
| 2011 | 3 | 5 | 13 | 24 | 3.48 | 2.7 | 10.729591 | -85.176164 | 2.491 | 0.044 |  |
| 2011 | 5 | 7 | 23 | 22 | 30.72 | 2.2 | 10.63367 | -85.00437 | 5.866 | 0.06 |  |
| 2011 | 5 | 7 | 23 | 25 | 5.24 | 2.2 | 10.635403 | -85.003849 | 5.881 | 0.065 |  |
| 2011 | 5 | 7 | 23 | 40 | 32.16 | 2.2 | 10.62862 | -85.000586 | 6.947 | 0.081 |  |
| 2011 | 5 | 8 | 2 | 7 | 3.12 | 2.2 | 10.638568 | -85.005957 | 6.008 | 0.067 |  |
| 2011 | 5 | 10 | 12 | 13 | 14.2 | 2 | 10.597983 | -84.95634 | 6.45 | 0.077 |  |
| 2011 | 5 | 10 | 12 | 27 | 55.44 | 2.8 | 10.59603 | -84.958301 | 6.593 | 0.065 |  |
| 2011 | 5 | 10 | 12 | 47 | 43.29 | 2.4 | 10.597102 | -84.959049 | 6.062 | 0.037 |  |
| 2011 | 5 | 10 | 13 | 3 | 26.57 | 2.2 | 10.594824 | -84.962069 | 6.469 | 0.045 |  |
| 2011 | 5 | 10 | 13 | 29 | 21.61 | 2.5 | 10.589757 | -84.964372 | 2.823 | 0.091 |  |
| 2011 | 5 | 11 | 2 | 29 | 21.23 | 2.3 | 10.596958 | -84.961662 | 6.624 | 0.05 |  |
| 2011 | 5 | 11 | 3 | 21 | 34.44 | 2.1 | 10.599493 | -84.957593 | 5.922 | 0.053 |  |
| 2011 | 5 | 11 | 10 | 44 | 27.14 | 2.4 | 10.583616 | -84.907788 | 5.064 | 0.037 |  |
| 2011 | 5 | 13 | 0 | 11 | 43.54 | 3 | 10.596031 | -84.957699 | 6.633 | 0.075 |  |
| 2011 | 5 | 13 | 6 | 12 | 1.11 | 3.1 | 10.60068 | -84.959049 | 6.6 | 0.056 |  |
| 2011 | 5 | 13 | 7 | 59 | 32.66 | 2.7 | 10.599007 | -84.959505 | 6.645 | 0.056 |  |
| 2011 | 5 | 22 | 17 | 16 | 42.12 | 2.4 | 10.596898 | -84.961149 | 6.655 | 0.066 |  |
| 2011 | 5 | 29 | 23 | 1 | 51.6 | 3.1 | 10.652344 | -85.091073 | 4.101 | 0.057 |  |
| 2011 | 6 | 11 | 22 | 55 | 7.1 | 2.7 | 10.693973 | -85.075741 | 6.223 | 0.044 |  |
| 2011 | 6 | 26 | 10 | 12 | 12.22 | 2.4 | 10.701462 | -85.087036 | 4.821 | 0.051 |  |
| 2011 | 6 | 26 | 10 | 20 | 13.78 | 2.5 | 10.697748 | -85.086654 | 4.928 | 0.052 |  |
| 2011 | 7 | 12 | 20 | 11 | 1.12 | 4.3 | 10.779245 | -85.089852 | 2.545 | 0.079 | 246.00 90.00 35.00 |
| 2011 | 7 | 12 | 21 | 5 | 42.48 | 2.8 | 10.755498 | -85.118953 | 6.281 | 0.085 |  |
| 2011 | 7 | 12 | 21 | 35 | 20.02 | 2.2 | 10.752813 | -85.132153 | 4.132 | 0.065 |  |
| 2011 | 7 | 12 | 22 | 8 | 20.4 | 2.9 | 10.753547 | -85.121468 | 5.589 | 0.076 |  |
| 2011 | 7 | 12 | 22 | 56 | 49 | 2.1 | 10.751863 | -85.122363 | 4.57 | 0.076 |  |
| 2011 | 7 | 12 | 23 | 13 | 53.4 | 1.7 | 10.750572 | -85.121053 | 3.925 | 0.051 |  |
| 2011 | 7 | 12 | 23 | 20 | 51.28 | 2.9 | 10.752569 | -85.124748 | 4.594 | 0.064 |  |
| 2011 | 7 | 12 | 23 | 30 | 12.48 | 1.7 | 10.753781 | -85.122518 | 7.925 | 0.094 |  |
| 2011 | 7 | 13 | 0 | 26 | 18.19 | 2.8 | 10.757901 | -85.130656 | 6.576 | 0.09 |  |
| 2011 | 7 | 13 | 1 | 9 | 17.95 | 2.7 | 10.734213 | -85.102214 | 4.46 | 0.059 |  |
| 2011 | 7 | 13 | 1 | 59 | 40.67 | 2.1 | 10.735607 | -85.093376 | 4.289 | 0.071 |  |
| 2011 | 7 | 13 | 13 | 42 | 52.81 | 2.4 | 10.695328 | -85.093359 | 5.868 | 0.033 |  |
| 2011 | 7 | 13 | 14 | 53 | 31.05 | 3.1 | 10.750694 | -85.128426 | 4.616 | 0.076 |  |
| 2011 | 7 | 13 | 16 | 44 | 28.94 | 2.7 | 10.750753 | -85.118872 | 3.96 | 0.085 |  |
| 2011 | 7 | 14 | 0 | 19 | 37.3 | 2.8 | 10.753102 | -85.122599 | 4.419 | 0.065 |  |
| 2011 | 7 | 14 | 9 | 16 | 14.01 | 2.2 | 10.752846 | -85.148812 | 5.347 | 0.1 |  |
| 2011 | 7 | 14 | 9 | 31 | 1.86 | 2.9 | 10.693979 | -85.089917 | 4.86 | 0.051 |  |
| 2011 | 7 | 14 | 10 | 10 | 39.05 | 2.6 | 10.746545 | -85.113729 | 4.651 | 0.074 |  |
| 2011 | 7 | 14 | 17 | 8 | 9.2 | 2.4 | 10.744895 | -85.126302 | 2.58 | 0.084 |  |
| 2011 | 7 | 15 | 19 | 50 | 2.12 | 2.6 | 10.698856 | -85.080265 | 6.152 | 0.047 |  |
| 2011 | 7 | 16 | 2 | 19 | 32.81 | 2.4 | 10.69486 | -85.075488 | 5.492 | 0.04 |  |
| 2011 | 7 | 16 | 15 | 11 | 12.21 | 2.2 | 10.701058 | -85.084692 | 4.503 | 0.038 |  |
| 2011 | 7 | 17 | 15 | 51 | 35.86 | 2.2 | 10.688536 | -85.09165 | 5.159 | 0.051 |  |
| 2011 | 7 | 17 | 16 | 3 | 53.08 | 2.1 | 10.685934 | -85.094491 | 5.903 | 0.073 |  |
| 2011 | 7 | 18 | 22 | 46 | 56.4 | 2.1 | 10.708824 | -85.079899 | 6.69 | 0.036 |  |
| 2011 | 7 | 18 | 22 | 47 | 22.8 | 2.3 | 10.705143 | -85.083423 | 6.515 | 0.035 |  |
| 2011 | 7 | 18 | 22 | 55 | 29.52 | 2.2 | 10.738794 | -85.103337 | 5.355 | 0.072 |  |
| 2011 | 7 | 19 | 23 | 55 | 34.44 | 2.1 | 10.73007 | -85.096191 | 9.398 | 0.062 |  |
| 2011 | 7 | 20 | 9 | 19 | 55.47 | 2.5 | 10.700031 | -85.084082 | 5.319 | 0.053 |  |
| 2011 | 7 | 21 | 4 | 7 | 11.8 | 2.6 | 10.697738 | -85.085905 | 4.895 | 0.041 |  |
| 2011 | 7 | 21 | 4 | 29 | 57.51 | 2.5 | 10.73724 | -85.098307 | 2.853 | 0.094 |  |
| 2011 | 7 | 29 | 8 | 25 | 3.89 | 2.8 | 10.594881 | -84.974422 | 3.236 | 0.092 |  |
| 2011 | 8 | 1 | 5 | 44 | 9.06 | 2.6 | 10.725483 | -85.114209 | 4.164 | 0.063 |  |
| 2011 | 8 | 1 | 8 | 10 | 44.57 | 2.6 | 10.748114 | -85.125505 | 4.227 | 0.059 |  |
| 2011 | 9 | 5 | 6 | 21 | 6.97 | 2.7 | 10.717672 | -85.105656 | 5.097 | 0.059 |  |
| 2011 | 9 | 24 | 13 | 34 | 2.41 | 2.2 | 10.695631 | -85.190552 | 3.867 | 0.077 |  |
| 2011 | 10 | 2 | 13 | 0 | 16.32 | 3.6 | 10.768101 | -85.142358 | 4.725 | 0.086 |  |
| 2011 | 10 | 2 | 13 | 59 | 30.1 | 3 | 10.766713 | -85.086019 | 4.596 | 0.082 |  |
| 2011 | 10 | 2 | 14 | 4 | 24.26 | 2.8 | 10.762531 | -85.086979 | 4.918 | 0.061 |  |
| 2011 | 10 | 2 | 20 | 32 | 3.96 | 3 | 10.758111 | -85.079598 | 5.647 | 0.045 |  |
| 2011 | 10 | 5 | 15 | 20 | 12.83 | 2.8 | 10.756433 | -85.097518 | 2.418 | 0.062 |  |
| 2011 | 10 | 22 | 1 | 48 | 45.82 | 2.8 | 10.763746 | -85.117179 | 7.694 | 0.066 |  |
| 2011 | 11 | 10 | 0 | 51 | 59.55 | 2.6 | 10.767234 | -85.127588 | 3.998 | 0.062 |  |
| 2011 | 11 | 18 | 23 | 46 | 56.34 | 3 | 10.773115 | -85.11141 | 6.054 | 0.072 |  |
| 2011 | 11 | 19 | 0 | 11 | 47.38 | 2.7 | 10.770353 | -85.116813 | 5.268 | 0.049 |  |
| 2011 | 11 | 24 | 10 | 32 | 20.21 | 2.7 | 10.78037 | -85.114762 | 5.907 | 0.078 |  |
| 2011 | 11 | 27 | 5 | 8 | 28.86 | 2.7 | 10.763263 | -85.10988 | 7.341 | 0.083 |  |
| 2011 | 12 | 5 | 2 | 45 | 34.34 | 2.1 | 10.706645 | -85.102466 | 6.866 | 0.062 |  |
| 2011 | 12 | 11 | 11 | 56 | 31.83 | 2.6 | 10.655651 | -85.101579 | 3.833 | 0.072 |  |
| 2012 | 1 | 12 | 6 | 42 | 55.56 | 2.7 | 10.772971 | -85.118937 | 6.377 | 0.059 |  |
| 2012 | 1 | 23 | 10 | 56 | 26.29 | 2.5 | 10.659839 | -85.089909 | 6.165 | 0.02 |  |
| 2012 | 2 | 1 | 16 | 26 | 17.24 | 2.6 | 10.767149 | -85.112174 | 7.072 | 0.078 |  |
| 2012 | 2 | 2 | 2 | 0 | 39.39 | 3 | 10.613125 | -84.988363 | 7.077 | 0.055 |  |
| 2012 | 2 | 2 | 9 | 57 | 39.63 | 2.8 | 10.766352 | -85.098771 | 2.115 | 0.076 |  |
| 2012 | 2 | 14 | 4 | 21 | 51.32 | 2.7 | 10.763036 | -85.114217 | 5.918 | 0.058 |  |
| 2012 | 2 | 21 | 11 | 3 | 18.02 | 3 | 10.606161 | -84.956991 | 5.497 | 0.088 |  |
| 2012 | 2 | 28 | 0 | 29 | 17.44 | 2.8 | 10.643329 | -85.01901 | 1.405 | 0.063 |  |
| 2012 | 4 | 1 | 13 | 49 | 2.9 | 3.3 | 10.666199 | -85.015031 | 4.086 | 0.082 |  |
| 2012 | 4 | 13 | 23 | 35 | 17.42 | 2.6 | 10.725736 | -85.092147 | 6.438 | 0.056 |  |
| 2012 | 4 | 22 | 2 | 9 | 59.35 | 2.5 | 10.60311 | -84.94515 | 7.042 | 0.062 |  |
| 2012 | 5 | 2 | 1 | 33 | 48.46 | 2.6 | 10.58495 | -84.929085 | 5.566 | 0.042 |  |
| 2012 | 5 | 23 | 4 | 46 | 56.6 | 2.8 | 10.613534 | -84.968083 | 3.85 | 0.059 |  |
| 2012 | 5 | 23 | 9 | 7 | 41.41 | 3.2 | 10.610389 | -84.968335 | 4.109 | 0.076 |  |
| 2012 | 5 | 30 | 8 | 43 | 34.35 | 3.1 | 10.741961 | -85.054476 | 5.283 | 0.101 |  |
| 2012 | 7 | 26 | 3 | 58 | 17.21 | 2.8 | 10.756973 | -85.068864 | 5.527 | 0.072 |  |
| 2012 | 7 | 26 | 4 | 46 | 17.57 | 3 | 10.752407 | -85.070996 | 5.465 | 0.067 |  |
| 2012 | 8 | 4 | 9 | 29 | 11.13 | 3.1 | 10.733526 | -85.16936 | 3.1 | 0.059 |  |
| 2012 | 9 | 5 | 17 | 57 | 56.52 | 2.9 | 10.596724 | -84.944604 | 3.445 | 0.08 |  |
| 2012 | 9 | 6 | 6 | 36 | 24.09 | 2.7 | 10.645166 | -85.055648 | 1.283 | 0.082 |  |
| 2012 | 9 | 6 | 10 | 47 | 34.52 | 3 | 10.603116 | -84.942879 | 2.914 | 0.09 |  |
| 2012 | 9 | 7 | 0 | 55 | 0.92 | 2.8 | 10.643028 | -85.05752 | 1.733 | 0.078 |  |
| 2012 | 9 | 7 | 5 | 2 | 36.81 | 2.1 | 10.598301 | -84.983065 | 6.307 | 0.042 |  |
| 2012 | 9 | 17 | 1 | 11 | 8.72 | 3.1 | 10.62621 | -84.998543 | 6.789 | 0.066 |  |
| 2012 | 9 | 17 | 6 | 58 | 0.09 | 2.8 | 10.655326 | -85.035775 | 4.901 | 0.048 |  |
| 2012 | 9 | 21 | 6 | 6 | 42.42 | 2.9 | 10.638967 | -84.973617 | 4.734 | 0.042 |  |
| 2012 | 9 | 22 | 0 | 15 | 54.28 | 2.9 | 10.641214 | -85.028337 | 2.214 | 0.054 |  |
| 2012 | 9 | 23 | 14 | 49 | 35.37 | 3.2 | 10.657467 | -84.993693 | 4.838 | 0.08 |  |
| 2012 | 9 | 23 | 19 | 26 | 31.92 | 2.8 | 10.659337 | -84.996037 | 4.402 | 0.056 |  |
| 2012 | 9 | 24 | 13 | 9 | 10.09 | 2.5 | 10.637522 | -84.975041 | 5.577 | 0.064 |  |
| 2012 | 9 | 27 | 0 | 23 | 30.64 | 2.4 | 10.586518 | -84.908374 | 2.674 | 0.08 |  |
| 2012 | 9 | 27 | 16 | 59 | 30.22 | 2.6 | 10.647147 | -85.026522 | 2.505 | 0.071 |  |
| 2012 | 9 | 27 | 18 | 19 | 0.98 | 2.8 | 10.652206 | -84.994181 | 4.559 | 0.078 |  |
| 2012 | 9 | 30 | 3 | 23 | 10.97 | 2.3 | 10.654564 | -84.995353 | 3.127 | 0.082 |  |
| 2012 | 10 | 1 | 18 | 57 | 1.12 | 3 | 10.761642 | -85.116081 | 7.142 | 0.055 |  |
| 2012 | 10 | 1 | 23 | 36 | 23 | 2.5 | 10.680412 | -85.013468 | 5.115 | 0.06 |  |
| 2012 | 10 | 2 | 7 | 30 | 2.55 | 2.3 | 10.656586 | -84.991471 | 4.788 | 0.058 |  |
| 2012 | 10 | 5 | 15 | 53 | 46.08 | 2.4 | 10.654569 | -84.995052 | 5.063 | 0.092 |  |
| 2012 | 10 | 6 | 4 | 28 | 40.64 | 2.4 | 10.68878 | -85.089608 | 5.717 | 0.061 |  |
| 2012 | 10 | 7 | 12 | 17 | 56.68 | 1.9 | 10.583529 | -85.002352 | 3.397 | 0.005 |  |
| 2012 | 10 | 9 | 19 | 7 | 54.68 | 2.4 | 10.638692 | -85.01377 | 2.577 | 0.039 |  |
| 2012 | 10 | 11 | 17 | 6 | 49.96 | 3.2 | 10.640517 | -85.014819 | 2.207 | 0.067 | 048.00 84.00 08.00 |
| 2012 | 10 | 16 | 20 | 44 | 47.94 | 2.2 | 10.587907 | -84.994507 | 7.618 | 0.063 |  |
| 2012 | 10 | 20 | 20 | 35 | 11.86 | 2.3 | 10.648225 | -85.050391 | 3.783 | 0.08 |  |
| 2012 | 10 | 21 | 4 | 44 | 52.65 | 3.2 | 10.600794 | -84.946354 | 8.835 | 0.072 |  |
| 2012 | 10 | 21 | 4 | 53 | 27.46 | 2.7 | 10.599685 | -84.945736 | 8.373 | 0.078 |  |
| 2012 | 10 | 21 | 5 | 2 | 40.51 | 2.9 | 10.598823 | -84.946932 | 8.597 | 0.075 | 180.00 45.00 -89.00 |
| 2012 | 10 | 21 | 6 | 28 | 15.22 | 2.2 | 10.597974 | -84.949154 | 8.714 | 0.053 |  |
| 2012 | 10 | 22 | 14 | 44 | 12.31 | 2.2 | 10.596816 | -84.969312 | 7.425 | 0.055 |  |
| 2012 | 10 | 22 | 15 | 18 | 40.73 | 1.9 | 10.595995 | -84.972046 | 6.584 | 0.036 |  |
| 2012 | 10 | 23 | 0 | 47 | 48.59 | 2.3 | 10.596134 | -84.971745 | 6.989 | 0.042 |  |
| 2012 | 10 | 28 | 6 | 34 | 10.6 | 2.4 | 10.611128 | -84.992619 | 5.848 | 0.067 |  |
| 2012 | 10 | 30 | 21 | 42 | 18.68 | 2.7 | 10.60456 | -84.944019 | 2.96 | 0.081 |  |
| 2012 | 11 | 2 | 3 | 45 | 34.19 | 2.7 | 10.658194 | -84.98514 | 2.067 | 0.079 |  |
| 2012 | 11 | 3 | 14 | 0 | 17.64 | 2.6 | 10.65562 | -84.977645 | 4.702 | 0.093 |  |
| 2012 | 11 | 3 | 16 | 54 | 5.96 | 3.1 | 10.637187 | -85.080623 | 3.746 | 0.067 |  |
| 2012 | 11 | 4 | 0 | 37 | 9.87 | 3.4 | 10.657215 | -84.983732 | 3.721 | 0.084 |  |
| 2012 | 11 | 5 | 1 | 22 | 30.08 | 2.5 | 10.664443 | -85.007397 | 5.088 | 0.09 |  |
| 2012 | 11 | 6 | 14 | 11 | 19.13 | 2.5 | 10.709084 | -85.058431 | 5.49 | 0.022 |  |
| 2012 | 11 | 7 | 8 | 37 | 50.17 | 2.2 | 10.594399 | -84.955241 | 6.165 | 0.038 |  |
| 2012 | 11 | 10 | 5 | 18 | 10.91 | 2.6 | 10.741884 | -85.178695 | 2.514 | 0.042 |  |
| 2012 | 11 | 23 | 0 | 59 | 30.35 | 3 | 10.62063 | -84.95953 | 3.508 | 0.086 |  |
| 2012 | 11 | 26 | 23 | 20 | 30.92 | 2.6 | 10.58018 | -84.925122 | 8.111 | 0.048 |  |
| 2012 | 11 | 30 | 0 | 45 | 6.99 | 2.2 | 10.656997 | -84.995874 | 5.412 | 0.085 |  |
| 2012 | 12 | 20 | 17 | 30 | 5.24 | 3.3 | 10.674143 | -85.006364 | 4.939 | 0.072 |  |
| 2012 | 12 | 24 | 9 | 3 | 37.55 | 3 | 10.485783 | -84.941829 | 4.849 | 0.048 |  |
| 2012 | 12 | 24 | 9 | 6 | 12.37 | 3 | 10.483065 | -84.941943 | 5.134 | 0.048 |  |
| 2013 | 1 | 21 | 8 | 15 | 32.92 | 2.9 | 10.669411 | -85.003687 | 4.762 | 0.105 |  |
| 2013 | 4 | 27 | 6 | 37 | 30.47 | 2.8 | 10.582393 | -84.990381 | 7.662 | 0.049 |  |
| 2013 | 6 | 2 | 23 | 25 | 53.44 | 2.8 | 10.766369 | -85.104313 | 6.535 | 0.063 |  |
| 2013 | 6 | 3 | 3 | 0 | 57.05 | 2.9 | 10.750158 | -85.121753 | 4.885 | 0.093 |  |
| 2013 | 6 | 5 | 4 | 46 | 41.56 | 2.9 | 10.760887 | -85.085026 | 4.72 | 0.056 |  |
| 2013 | 6 | 7 | 1 | 3 | 32.1 | 2.6 | 10.729522 | -85.151676 | 4.11 | 0.04 |  |
| 2013 | 6 | 21 | 22 | 17 | 51.22 | 2.7 | 10.602996 | -84.962891 | 6.693 | 0.079 |  |
| 2013 | 7 | 10 | 1 | 44 | 12.03 | 2.9 | 10.578137 | -84.927677 | 4.652 | 0.065 |  |
| 2013 | 7 | 12 | 15 | 29 | 6.46 | 2.8 | 10.580237 | -84.925618 | 4.769 | 0.071 |  |
| 2013 | 7 | 21 | 3 | 44 | 26 | 2.9 | 10.755628 | -85.079761 | 5.74 | 0.065 |  |
| 2013 | 8 | 20 | 20 | 17 | 30.38 | 3.2 | 10.61476 | -84.970093 | 6.249 | 0.076 |  |
| 2013 | 8 | 22 | 23 | 4 | 57.96 | 2.2 | 10.711718 | -85.178784 | 0.285 | 0.128 |  |
| 2013 | 8 | 23 | 4 | 56 | 29.37 | 3 | 10.604298 | -84.9646 | 5.512 | 0.077 |  |
| 2013 | 8 | 29 | 6 | 57 | 18.94 | 3 | 10.62328 | -84.976742 | 6.306 | 0.084 | 201.00 40.00 -25.00 |
| 2013 | 8 | 29 | 9 | 9 | 57.13 | 3.1 | 10.699355 | -85.069377 | 4.939 | 0.065 |  |
| 2013 | 9 | 1 | 3 | 9 | 32.17 | 2.8 | 10.668859 | -84.994035 | 2.686 | 0.111 |  |
| 2013 | 9 | 6 | 6 | 25 | 26.61 | 3.1 | 10.729814 | -85.036369 | 5.037 | 0.081 | 078.00 81.70 -23.66 |
| 2013 | 9 | 7 | 9 | 42 | 43.9 | 3 | 10.731063 | -85.040796 | 4.464 | 0.099 |  |
| 2013 | 9 | 8 | 7 | 33 | 1.77 | 3 | 10.692424 | -85.019751 | 6.047 | 0.066 |  |
| 2013 | 9 | 8 | 7 | 35 | 52.6 | 2.7 | 10.691556 | -85.020972 | 6.024 | 0.068 | 345.00 40.00 -89.00 |
| 2013 | 9 | 13 | 3 | 31 | 21.67 | 2.9 | 10.666243 | -84.997721 | 5 | 0.09 | 011.00 85.00 30.00 |
| 2013 | 9 | 15 | 2 | 25 | 55.33 | 2.6 | 10.698895 | -85.070418 | 5.049 | 0.055 |  |
| 2013 | 9 | 28 | 1 | 41 | 37.59 | 3.2 | 10.613528 | -84.953524 | 5.097 | 0.077 |  |
| 2013 | 10 | 4 | 0 | 22 | 17.2 | 2.8 | 10.617647 | -84.972599 | 4.011 | 0.075 |  |
| 2013 | 10 | 5 | 0 | 36 | 25.49 | 2.9 | 10.618555 | -84.972176 | 3.878 | 0.076 |  |
| 2013 | 10 | 5 | 4 | 21 | 49.39 | 2.8 | 10.62037 | -84.97015 | 2.348 | 0.084 |  |
| 2013 | 10 | 21 | 11 | 49 | 51.03 | 3.1 | 10.845693 | -85.148193 | 4.006 | 0.043 |  |
| 2013 | 11 | 10 | 19 | 4 | 4.8 | 2.7 | 10.770568 | -85.089722 | 6.859 | 0.07 |  |
| 2013 | 11 | 21 | 1 | 17 | 6.14 | 3 | 10.666665 | -85.010474 | 2.381 | 0.069 |  |
| 2013 | 12 | 3 | 5 | 40 | 25.78 | 3.1 | 10.726056 | -85.174536 | 2.591 | 0.059 |  |
| 2014 | 1 | 21 | 8 | 41 | 27.41 | 2.4 | 10.510506 | -84.959692 | 4.965 | 0.07 |  |
| 2014 | 1 | 21 | 12 | 29 | 10.02 | 2.7 | 10.711605 | -85.021183 | 5.644 | 0.065 |  |
| 2014 | 1 | 23 | 23 | 36 | 53.62 | 2.7 | 10.515784 | -84.960254 | 4.525 | 0.07 |  |
| 2014 | 2 | 3 | 7 | 32 | 34.66 | 2.9 | 10.679352 | -85.010832 | 5.785 | 0.091 |  |
| 2014 | 2 | 4 | 1 | 33 | 15.8 | 2.8 | 10.624654 | -84.972314 | 7.474 | 0.063 |  |
| 2014 | 2 | 26 | 4 | 36 | 51.27 | 2.7 | 10.682837 | -85.180876 | 4.64 | 0.075 |  |
| 2014 | 2 | 26 | 12 | 10 | 13.35 | 2.8 | 10.685528 | -85.179948 | 4.691 | 0.057 |  |
| 2014 | 2 | 26 | 14 | 11 | 0.34 | 2.8 | 10.689902 | -85.180363 | 3.846 | 0.069 |  |
| 2014 | 2 | 27 | 7 | 24 | 20.51 | 3.1 | 10.61754 | -84.96709 | 5.028 | 0.078 |  |
| 2014 | 3 | 11 | 3 | 25 | 18.92 | 2.6 | 10.637979 | -85.036971 | 2.563 | 0.094 |  |
| 2014 | 3 | 11 | 5 | 39 | 48.13 | 2.9 | 10.649542 | -85.040544 | 3.214 | 0.071 |  |
| 2014 | 3 | 11 | 8 | 12 | 18.33 | 2.9 | 10.651631 | -85.040828 | 3.325 | 0.073 |  |
| 2014 | 3 | 12 | 15 | 1 | 34.58 | 3.2 | 10.803033 | -85.134725 | 3.309 | 0.081 | 119.00 65.00 -78.00 |
| 2014 | 3 | 12 | 19 | 44 | 30 | 3.2 | 10.753784 | -85.087443 | 3.531 | 0.065 |  |
| 2014 | 3 | 26 | 1 | 46 | 5.13 | 2.9 | 10.771417 | -85.079142 | 8.783 | 0.07 | 195.00 56.00 -71.00 |
| 2014 | 3 | 27 | 18 | 10 | 22.06 | 3.1 | 10.770453 | -85.117399 | 6.374 | 0.061 |  |
| 2014 | 4 | 10 | 19 | 7 | 36.22 | 3.1 | 10.574307 | -84.917017 | 6.339 | 0.079 |  |
| 2014 | 4 | 10 | 19 | 16 | 13.5 | 2.9 | 10.589488 | -84.929614 | 4.324 | 0.061 |  |
| 2014 | 4 | 28 | 11 | 8 | 13.18 | 3 | 10.621917 | -84.976636 | 3.717 | 0.071 |  |
| 2014 | 4 | 30 | 11 | 59 | 30.44 | 2.9 | 10.736431 | -85.169189 | 3.646 | 0.037 |  |
| 2014 | 5 | 12 | 17 | 22 | 49.28 | 3.1 | 10.597656 | -84.93549 | 2.591 | 0.045 |  |
| 2014 | 5 | 25 | 12 | 1 | 13.36 | 3 | 10.664704 | -84.993351 | 5.288 | 0.076 |  |
| 2014 | 5 | 29 | 6 | 43 | 37.88 | 2.6 | 10.71177 | -85.211572 | 3.932 | 0.064 |  |
| 2014 | 5 | 29 | 10 | 20 | 8.64 | 2.8 | 10.685582 | -85.098039 | 5.051 | 0.06 |  |
| 2014 | 5 | 30 | 3 | 22 | 58.87 | 2.5 | 10.602425 | -84.961442 | 5.69 | 0.056 |  |
| 2014 | 5 | 30 | 3 | 24 | 27.26 | 2.5 | 10.605212 | -84.96792 | 3.974 | 0.07 |  |
| 2014 | 6 | 4 | 21 | 37 | 22.04 | 3 | 10.609825 | -84.964437 | 7.561 | 0.087 |  |
| 2014 | 6 | 7 | 8 | 57 | 52.03 | 2.7 | 10.738884 | -85.171216 | 3.673 | 0.06 |  |
| 2014 | 6 | 13 | 1 | 59 | 30.67 | 2.5 | 10.782892 | -85.124772 | 3.44 | 0.071 |  |
| 2014 | 6 | 16 | 4 | 31 | 49.68 | 2.6 | 10.689325 | -85.201807 | 2.81 | 0.068 |  |
| 2014 | 6 | 17 | 3 | 55 | 37.51 | 2.9 | 10.675339 | -85.205697 | 2.387 | 0.062 |  |
| 2014 | 6 | 30 | 15 | 35 | 19.09 | 3 | 10.765504 | -85.110872 | 7.132 | 0.058 |  |
| 2014 | 7 | 12 | 21 | 44 | 43.34 | 3.1 | 10.776037 | -85.120011 | 5.099 | 0.09 |  |
| 2014 | 7 | 13 | 17 | 18 | 35.74 | 3.1 | 10.751424 | -85.072811 | 4.712 | 0.036 |  |
| 2014 | 7 | 19 | 7 | 10 | 12.67 | 2.9 | 10.802317 | -85.129948 | 4.603 | 0.037 |  |
| 2014 | 7 | 24 | 11 | 41 | 17.31 | 3 | 10.782827 | -85.119637 | 1.314 | 0.009 |  |
| 2014 | 8 | 2 | 20 | 17 | 16.36 | 2.8 | 10.632998 | -84.981616 | 4.711 | 0.097 |  |
| 2014 | 8 | 2 | 23 | 47 | 27.64 | 2.4 | 10.632719 | -84.982902 | 5.106 | 0.081 |  |
| 2014 | 8 | 3 | 0 | 8 | 6.37 | 2.7 | 10.63211 | -84.977979 | 4.688 | 0.056 |  |
| 2014 | 8 | 3 | 2 | 51 | 22.16 | 2 | 10.634176 | -84.982414 | 7.136 | 0.044 |  |
| 2014 | 8 | 3 | 3 | 31 | 52.5 | 3.1 | 10.630889 | -84.979598 | 5.736 | 0.084 |  |
| 2014 | 8 | 3 | 4 | 4 | 32.46 | 2.2 | 10.63304 | -84.979769 | 4.411 | 0.059 |  |
| 2014 | 8 | 3 | 4 | 22 | 25.21 | 2.9 | 10.633158 | -84.980615 | 4.948 | 0.07 |  |
| 2014 | 8 | 3 | 4 | 27 | 57.71 | 1.1 | 10.632629 | -84.980143 | 4.762 | 0.071 |  |
| 2014 | 8 | 3 | 5 | 49 | 11.6 | 3.2 | 10.630312 | -84.980062 | 5.864 | 0.078 |  |
| 2014 | 8 | 3 | 6 | 19 | 40.78 | 2.4 | 10.630821 | -84.980412 | 5.902 | 0.051 |  |
| 2014 | 8 | 25 | 5 | 52 | 11.81 | 2.1 | 10.642207 | -84.987996 | 2.537 | 0.108 |  |
| 2014 | 9 | 1 | 15 | 33 | 5.01 | 2.3 | 10.690804 | -85.019824 | 6.084 | 0.059 |  |
| 2014 | 9 | 9 | 7 | 40 | 31.21 | 1.8 | 10.627453 | -84.954077 | 5.719 | 0.052 |  |
| 2014 | 9 | 17 | 21 | 27 | 52.5 | 2.6 | 10.681129 | -85.013525 | 5.773 | 0.077 |  |
| 2014 | 10 | 7 | 7 | 29 | 29.35 | 2.3 | 10.65566 | -84.99633 | 4.236 | 0.086 |  |
| 2014 | 10 | 15 | 3 | 46 | 30.32 | 1.7 | 10.681582 | -85.017065 | 5.558 | 0.056 |  |
| 2014 | 10 | 15 | 3 | 46 | 50.64 | 2.2 | 10.681081 | -85.016488 | 6.001 | 0.067 |  |
| 2014 | 10 | 16 | 19 | 32 | 12.16 | 3.5 | 10.597105 | -84.940934 | 6.781 | 0.08 |  |
| 2014 | 11 | 17 | 16 | 7 | 21.27 | 2.6 | 10.65897 | -84.997965 | 5.8 | 0.095 |  |
| 2014 | 12 | 1 | 11 | 40 | 43.97 | 2.4 | 10.585269 | -84.970028 | 3 | 0.041 |  |
| 2014 | 12 | 3 | 17 | 4 | 19.44 | 2.8 | 10.721337 | -85.023576 | 5.457 | 0.068 |  |
| 2014 | 12 | 22 | 8 | 53 | 16.32 | 1.9 | 10.607356 | -84.945207 | 4.882 | 0.073 |  |
| 2014 | 12 | 30 | 15 | 18 | 28.97 | 2.7 | 10.621922 | -84.970492 | 4.847 | 0.081 |  |
| 2014 | 12 | 31 | 5 | 14 | 7.26 | 2.4 | 10.622451 | -84.970752 | 4.662 | 0.078 |  |
| 2015 | 1 | 8 | 20 | 26 | 40.68 | 2.8 | 10.578819 | -84.879834 | 5.878 | 0.046 |  |
| 2015 | 1 | 8 | 22 | 12 | 27.12 | 2.4 | 10.59247 | -84.886353 | 6.083 | 0.046 |  |
| 2015 | 1 | 17 | 23 | 1 | 46.42 | 2.6 | 10.76728 | -85.101196 | 6.864 | 0.055 |  |
| 2015 | 2 | 15 | 7 | 55 | 54.23 | 3.4 | 10.696236 | -85.024683 | 1.882 | 0.085 |  |
| 2015 | 3 | 8 | 4 | 28 | 14.3 | 2.6 | 10.598582 | -84.948983 | 7.176 | 0.059 |  |
| 2015 | 3 | 17 | 2 | 43 | 38.17 | 2.8 | 10.584097 | -84.929378 | 6.561 | 0.056 |  |
| 2015 | 3 | 23 | 1 | 50 | 22.95 | 2.8 | 10.768214 | -85.111938 | 6.492 | 0.053 |  |
| 2015 | 5 | 3 | 0 | 45 | 14.92 | 2.4 | 10.6306 | -84.981396 | 5.771 | 0.055 |  |
| 2015 | 5 | 6 | 2 | 59 | 51.22 | 2 | 10.58535 | -84.933089 | 4.804 | 0.055 |  |
| 2015 | 5 | 9 | 12 | 8 | 20.86 | 2.2 | 10.438192 | -85.055111 | 6.207 | 0.249 |  |
| 2015 | 5 | 13 | 10 | 13 | 39.67 | 3.1 | 10.430235 | -85.054858 | 7.46 | 0.249 |  |
| 2015 | 6 | 1 | 16 | 28 | 53.38 | 2.4 | 10.672857 | -84.993766 | 4.654 | 0.072 |  |
| 2015 | 6 | 12 | 23 | 51 | 1.96 | 3.2 | 10.739137 | -85.173494 | 2.634 | 0.066 |  |
| 2015 | 6 | 12 | 23 | 51 | 16.68 | 3.1 | 10.735105 | -85.174463 | 2.481 | 0.073 |  |
| 2015 | 6 | 12 | 23 | 55 | 4.58 | 2.7 | 10.739347 | -85.174748 | 2.606 | 0.086 |  |
| 2015 | 7 | 5 | 2 | 24 | 17.57 | 2.8 | 10.599229 | -84.950936 | 7.619 | 0.063 |  |
| 2015 | 7 | 5 | 2 | 24 | 17.57 | 3.1 | 10.599659 | -84.950627 | 7.642 | 0.054 |  |
| 2015 | 7 | 8 | 6 | 18 | 46.09 | 3.3 | 10.724667 | -85.172502 | 2.707 | 0.06 |  |
| 2015 | 7 | 8 | 6 | 18 | 46.09 | 3.1 | 10.724754 | -85.172192 | 2.696 | 0.057 |  |
| 2015 | 7 | 8 | 6 | 42 | 15.86 | 3.1 | 10.72226 | -85.173853 | 2.518 | 0.063 |  |
| 2015 | 7 | 8 | 6 | 42 | 15.85 | 3.1 | 10.722497 | -85.173828 | 2.522 | 0.061 |  |
| 2015 | 7 | 8 | 6 | 48 | 22.73 | 3.1 | 10.722882 | -85.174251 | 2.579 | 0.076 |  |
| 2015 | 7 | 8 | 6 | 48 | 22.74 | 3.1 | 10.722854 | -85.174162 | 2.532 | 0.06 |  |
| 2015 | 7 | 8 | 6 | 58 | 30.66 | 2.7 | 10.723292 | -85.173535 | 1.981 | 0.059 |  |
| 2015 | 7 | 8 | 7 | 7 | 24.12 | 2.6 | 10.720256 | -85.173088 | 2.496 | 0.076 |  |
| 2015 | 7 | 13 | 11 | 56 | 6.28 | 2.8 | 10.664697 | -84.992448 | 5.059 | 0.069 |  |
| 2015 | 7 | 19 | 5 | 32 | 14.22 | 2.6 | 10.739782 | -85.105949 | 5.761 | 0.092 |  |
| 2015 | 7 | 22 | 9 | 8 | 50.26 | 2.7 | 10.751978 | -85.069727 | 4.853 | 0.064 |  |
| 2015 | 8 | 2 | 17 | 51 | 0.4 | 2.5 | 10.580526 | -84.926782 | 4.904 | 0.064 |  |
| 2015 | 9 | 29 | 18 | 57 | 26.36 | 2.7 | 10.60545 | -84.826294 | 6.761 | 0.074 |  |
| 2015 | 9 | 29 | 23 | 46 | 57.84 | 2.6 | 10.605449 | -84.830111 | 6.879 | 0.074 |  |
| 2015 | 10 | 18 | 4 | 7 | 45.67 | 2.6 | 10.61501 | -84.98995 | 5.303 | 0.072 |  |
| 2015 | 10 | 18 | 13 | 4 | 6.68 | 2.3 | 10.609807 | -84.991805 | 5.971 | 0.067 |  |
| 2015 | 10 | 27 | 10 | 33 | 59.19 | 3.2 | 10.631541 | -84.967358 | 7.371 | 0.093 |  |
| 2015 | 10 | 27 | 15 | 40 | 9.71 | 2.3 | 10.673209 | -85.215348 | 1.043 | 0.121 |  |
| 2015 | 10 | 29 | 7 | 49 | 5.3 | 3.6 | 10.73799 | -85.170955 | 2.901 | 0.061 |  |
| 2015 | 11 | 11 | 4 | 13 | 11.42 | 2.2 | 10.715859 | -85.055998 | 6.322 | 0.074 |  |
| 2015 | 11 | 13 | 0 | 47 | 10.37 | 2.3 | 10.714688 | -85.026229 | 6.697 | 0.056 |  |
| 2015 | 11 | 18 | 18 | 36 | 0.24 | 2.3 | 10.585807 | -84.910579 | 8.918 | 0.075 |  |
| 2015 | 12 | 4 | 14 | 59 | 33.08 | 3 | 10.697003 | -85.056657 | 5.469 | 0.062 | 008.00 80.00 -74.00 |
| 2015 | 12 | 4 | 16 | 3 | 59.51 | 2.5 | 10.698858 | -85.0552 | 5.764 | 0.041 |  |
| 2015 | 12 | 4 | 16 | 12 | 21.21 | 3.6 | 10.696641 | -85.056185 | 5.339 | 0.061 |  |
| 2015 | 12 | 4 | 23 | 34 | 49.72 | 3.1 | 10.695048 | -85.055428 | 5.646 | 0.05 |  |
| 2015 | 12 | 13 | 17 | 46 | 19.46 | 3 | 10.698077 | -85.200187 | 1.403 | 0.075 |  |
| 2015 | 12 | 21 | 16 | 25 | 5.41 | 2.9 | 10.668901 | -85.003149 | 3.955 | 0.067 |  |
| 2015 | 12 | 25 | 12 | 49 | 35.93 | 3 | 10.772074 | -85.086955 | 6.285 | 0.065 |  |
| 2015 | 12 | 30 | 16 | 2 | 48.01 | 3.3 | 10.76358 | -85.081372 | 4.179 | 0.054 |  |
| 2016 | 1 | 5 | 9 | 6 | 43.66 | 2.7 | 10.824993 | -85.149471 | 3.987 | 0.043 |  |
| 2016 | 1 | 7 | 9 | 15 | 48.43 | 2.7 | 10.678406 | -85.202962 | 1.54 | 0.083 |  |
| 2016 | 1 | 11 | 17 | 28 | 27.3 | 2.5 | 10.768759 | -85.087817 | 5.886 | 0.061 |  |
| 2016 | 1 | 13 | 20 | 52 | 25.38 | 2.3 | 10.75162 | -85.071826 | 5.165 | 0.05 |  |
| 2016 | 1 | 31 | 1 | 4 | 6.1 | 2.6 | 10.604662 | -84.967708 | 5.195 | 0.042 |  |
| 2016 | 2 | 16 | 8 | 5 | 16.91 | 2.6 | 10.66007 | -84.991024 | 6.302 | 0.083 |  |
| 2016 | 3 | 16 | 13 | 43 | 22.58 | 2.2 | 10.701283 | -85.017155 | 6.297 | 0.098 |  |
| 2016 | 3 | 26 | 5 | 15 | 31.73 | 2.6 | 10.635507 | -84.980762 | 1.444 | 0.091 |  |
| 2016 | 3 | 26 | 15 | 23 | 57.32 | 2.4 | 10.692164 | -85.104867 | 3.926 | 0.101 |  |
| 2016 | 4 | 1 | 2 | 0 | 54.83 | 2.3 | 10.703786 | -85.029403 | 3.403 | 0.077 |  |
| 2016 | 7 | 3 | 1 | 58 | 29.87 | 5.4 | 10.759848 | -85.062573 | 1.882 | 0.08 | 230.00 89.00 05.00 |
| 2016 | 7 | 3 | 2 | 3 | 14.39 | 4 | 10.763031 | -85.066781 | 4.248 | 0.053 |  |
| 2016 | 7 | 3 | 2 | 16 | 12.14 | 5.1 | 10.756398 | -85.061857 | 8.069 | 0.057 | 191.00 40.00 -81.00 |
| 2016 | 7 | 3 | 2 | 43 | 30.73 | 2.7 | 10.758982 | -85.074919 | 2.928 | 0.068 |  |
| 2016 | 7 | 3 | 2 | 44 | 37.6 | 2.5 | 10.748263 | -85.059644 | 2.654 | 0.06 |  |
| 2016 | 7 | 3 | 3 | 35 | 34.63 | 2.6 | 10.813774 | -85.10507 | 2.895 | 0.003 |  |
| 2016 | 7 | 3 | 4 | 27 | 33.51 | 3.6 | 10.731268 | -85.04707 | 1.806 | 0.058 |  |
| 2016 | 7 | 3 | 4 | 51 | 38.92 | 2.6 | 10.780226 | -85.086914 | 3.802 | 0.059 |  |
| 2016 | 7 | 3 | 5 | 38 | 55.65 | 3.9 | 10.795491 | -85.105509 | 2.655 | 0.052 | 232.00 75.00 00.00 |
| 2016 | 7 | 3 | 8 | 22 | 39.73 | 2.8 | 10.756191 | -85.05988 | 8.418 | 0.057 |  |
| 2016 | 7 | 5 | 4 | 48 | 11.79 | 3 | 10.775554 | -85.104753 | 3.296 | 0.037 |  |
| 2016 | 7 | 20 | 8 | 6 | 45.33 | 3.8 | 10.72439 | -85.039453 | 0.728 | 0.074 |  |
| 2016 | 7 | 24 | 3 | 11 | 41.04 | 2.8 | 10.723588 | -85.035417 | 1.814 | 0.074 |  |
| 2016 | 7 | 29 | 4 | 21 | 2.63 | 2.2 | 10.736349 | -85.04974 | 4.231 | 0.06 |  |

1 Waldhauser, F. & Ellsworth, W. L. A double-difference earthquake location algorithm: Method and application to the northern Hayward fault. *Bulletin of the Seismological Society of America* **90**, 1353-1368 (2000).

2 Snoke, J. A., Munsey, J. W., Teague, A. G. & Bollinger, G. A. A program for focal mechanism determination by combined use of polarity and SV-P amplitude ratio data. *Earthquake Notes* **55**, 15-20 (1984).
